# Systematic cryopreservation study of cardiac myoblasts in suspension

**DOI:** 10.1371/journal.pone.0295131

**Published:** 2024-03-06

**Authors:** Elham Ashrafi, Milica Radisic, Janet A. W. Elliott

**Affiliations:** 1 Department of Chemical and Materials Engineering, University of Alberta, Edmonton, Alberta, Canada; 2 Institute of Biomedical Engineering, University of Toronto, Toronto, Ontario, Canada; 3 Department of Chemical Engineering and Applied Chemistry, University of Toronto, Toronto, Ontario, Canada; 4 Department of Laboratory Medicine and Pathology, University of Alberta, Edmonton, Alberta, Canada; Cardiff Metropolitan University, UNITED KINGDOM

## Abstract

H9c2 myoblasts are a cell line derived from embryonic rat heart tissue and demonstrate the ability to differentiate to cardiac myotubes upon reduction of the serum concentration (from 10% to 1%) and addition of all-trans retinoic acid in the growth medium. H9c2 cells are increasingly being used as an easy-to-culture proxy for some functions of cardiomyocytes. The cryobiology of cardiac cells including H9c2 myoblasts has not been studied as extensively as that of some cell types. Consequently, it is important to characterize the cryobiological response and systematically develop well-optimized cryopreservation protocols for H9c2 cells to have optimal and consistent viability and functionality after thaw for high quality studies with this cell type. In this work, an interrupted slow cooling protocol (graded freezing) was applied to characterize H9c2 response throughout the cooling profile. Important factors that affect the cell response were examined, and final protocols that provided the highest post-thaw viability are reported. One protocol uses the common cryoprotectant dimethyl sulfoxide combined with hydroxyethyl starch, which will be suitable for applications in which the presence of dimethyl sulfoxide is not an issue; and the other protocol uses glycerol as a substitute when there is a desire to avoid dimethyl sulfoxide. Both protocols achieved comparable post-thaw viabilities (higher than 80%) based on SYTO 13/GelRed flow cytometry results. H9c2 cells cryopreserved by either protocol showed ability to differentiate to cardiac myotubes comparable to fresh (unfrozen) H9c2 cells, and their differentiation to cardiac myotubes was confirmed with *i*) change in cell morphology, *ii*) expression of cardiac marker troponin I, and *iii*) increase in mitochondrial mass.

## Introduction

Heart disease is one of the major causes of death in Canada and the United States [1,2]. Cardiovascular disease costs the Canadian economy more than $21.2 billion every year in health care services and medicine costs [3]. The high frequency of cardiovascular disease—including heart failure—necessitates the availability of model platforms (e.g., cardiomyocytes and/or engineered heart tissues) to *i*) study heart disease; *ii*) investigate the efficacy of heart drugs; and *iii*) assess cardiotoxicity of other drugs. Thus, it would be beneficial to develop optimized cryopreservation protocols for a variety of cardiac cells and tissues to enable distribution of cells for research. The cryobiology of cardiac cells has not been studied as extensively as that of some cell types.

The main advantages of cryopreservation are *i*) providing on-demand access for clinical use or research; *ii*) facilitating transportation; *iii*) long-term storage; *iv*) minimizing the variabilities in outcomes of research compared to fresh samples; and *v*) lowering the cost. Cells experience several stresses during cryopreservation and therefore, successful cryopreservation depends on several parameters including cooling rate, warming rate, and the types and concentrations of cryoprotectants, the optimal selection of which are different for different cell types. Characterizing the cell response throughout the cooling profile provides understanding required to efficiently find an optimal protocol that yields high post-thaw viability and function. The cell response to freeze/thaw stresses significantly depends on the properties of the cell membrane, including water permeability that regulates the transport of water entering and exiting the cell during cooling and warming. The cell membrane is thought to be the primary site of freezing injury [4–6]. Hence, membrane integrity is a fundamental measure of cell response to freeze/thaw stress and, accordingly, has been used to assess the cell response to freeze/thaw processes during optimization steps.

Mazur established the well-known two-factor hypothesis of cryoinjury to describe interpretations of optimal cooling rates whereby two independent mechanisms of cryoinjury impose stress on cells [7]. The first mechanism defines damage caused by cooling rates slower than the cell optimal rate. When a cell suspension is cooled slowly to temperatures below 0°C (the freezing point of an isotonic solution is −0.6°C [8]), ice first forms extracellularly, either spontaneously or by purposeful nucleation. The reasons why the ice forms externally first and not intracellularly are that *i*) the volume of the extracellular region is much larger than individual intracellular volumes, and because ice formation is a stochastic phenomenon, the probability of ice formation in a larger volume is much higher; and *ii*) there are more nucleators outside the cells [8]. As ice forms outside the cells, the concentration of extracellular solutes in the remaining unfrozen fraction increases. The plasma membrane prevents the growth of ice crystals from the extracellular environment into the cell interior above −10°C [5] due to the Gibbs–Thomson effect whereby ice requires a lower temperature to grow when the ice–solution interface is highly curved by the requirement of growing through small membrane pores [9–11]. Therefore, the intracellular water becomes supercooled even in the presence of external ice. The supercooled intracellular water flows out of the cell and freezes externally because it has a higher chemical potential than that of water in the partly frozen extracellular medium. In this case, cells dehydrate significantly. Dehydration itself is a source of cell injury, for example, by bringing structural proteins into contact and causing protein denaturation [12]. In addition, cells may be damaged by solute toxicity caused by prolonged exposure to high concentrations of solutes both extracellularly and intracellularly [13]. Slow-cooling injury is also called solution effects injury [14,15].

The second mechanism describes the damage caused by cooling cells at cooling rates faster than the optimal rate. When cooling is fast, the intracellular water does not have adequate time to depart the cell, and subsequently, cells freeze internally. The presence of large intracellular ice crystals is lethal [11,16]. Cryopreservation protocols rely on optimum cooling rates that are sufficiently rapid to avoid extended exposure to high concentrations of solutes, and still sufficiently slow to decrease the probability of intracellular ice formation [17].

An adequate number of cells do not survive even at optimal cooling rates. Accordingly, cryoprotective agents are used to provide additional protection from freeze/thaw stresses. Choosing the most suitable cryoprotectants is very important in cell cryopreservation. Cryoprotectants are categorized in two major groups (permeating and non-permeating cryoprotectants). Permeating cryoprotectants, which protect cells against slow cooling injuries, can penetrate the cells. Dimethyl sulfoxide (Me_2_SO), and glycerol (when sufficient time is provided for glycerol to permeate the cells at room temperature [18,19]) are examples of permeating cryoprotectants that protect cells by depressing the freezing point (colligatively), which decreases the amount of extracellular ice formed at each temperature, and reduces solute concentrations during freezing. Generally speaking, permeating cryoprotectants increase the intracellular and extracellular osmolality, and reduce the level of cell shrinkage [20,21]. Non-permeating cryoprotectants such as hydroxyethyl starch (HES) [22] (and glycerol when sufficient time is not provide for permeation [18,23]), which protect cells against rapid cooling injuries, are unable to penetrate cells. They protect cells by increasing extracellular osmolality, osmotically drawing water from cells at higher sub-zero temperatures where the concentration of solutes is not that high yet; thus, sufficient water leaves the cell and the probability of intracellular ice formation decreases [18,23–25]. Addition of HES does not improve slow cooling results significantly; however, it improves the rapid cooling results because of its ability to protect cells against intracellular ice formation during plunging into liquid nitrogen [18,23,26].

Cryoprotectant toxicity is an important limiting factor for the successful cryopreservation of living cells [27–29]; therefore, minimizing the toxicity of cryoprotectants is crucial to cryopreserving many cells. Cryoprotectant toxicity can be mitigated by using lower concentrations [30], shorter exposure times, and/or lower temperatures [31]. When lower concentrations of permeating cryoprotectants are used, addition of a non-permeating cryoprotectant has been shown to be beneficial [26,32–39]. The main reason is that when a non-permeating cryoprotectant (such as HES) is used in addition to Me_2_SO, HES promotes the water efflux from inside the cell at higher sub-zero temperatures (between −10°C to −20°C) when the other solutes are less concentrated compared to when HES is not present; accordingly, HES reduces the amount of intracellular ice formation during plunging into liquid nitrogen [18]. Among different combinations of Me_2_SO and HES, 5% Me_2_SO and 6% HES has been shown to be the most effective combination by previous studies for blood stem cells [34,40–45], human granulocytes [37], bone marrow [46], monocytes [47], canine bone marrow [48], baboon granulocytes [49], and guinea pig granulocytes [50]. Me_2_SO has been reported to be toxic for neurons and astrocytes [51,52] even at very small concentrations. Accordingly, another solution to avoid the toxicity of Me_2_SO altogether is to consider glycerol as a far less toxic alternative. Glycerol is used as a moisturizing, solvent, and lubricating agent in many personal care products such as toothpaste, mouthwashes, and soaps as well as in the pharmaceutical industry to prevent the drying of creams and lotions. Glycerol as a cryoprotectant was first discovered by Polge, Smith, & Parks [53] in 1949 and has been used in many studies so far [18,23,54–60]. Glycerol has been shown to act as both a permeating and a non-permeating cryoprotectant depending on the time and temperature of exposure. Studies have shown that glycerol acts as a non-permeating cryoprotectant at lower temperatures (for example 0°C) and/or with shorter incubation times [18,23,55]. On the other hand, glycerol acts as a permeating cryoprotectant at higher temperatures (room temperature) and longer incubation times [18,23,56,57,59,60].

Many researchers have tried to cryopreserve cardiac cells over the years, although the results reported by scientists regarding cardiac post-thaw viability and functionality are variable. For example, cardiac cell cryopreservation studies were started by Schopf–Ebner et al. [61]. They reported that only 8% of chick embryo heart cells survived after thaw, and only a small percentage of these surviving cells (5–20%) were beating. Also, the beating rhythm of cryopreserved cells increased significantly such that most of the cells even burst. Later, Yokomuro et al. [62] investigated the effects of cryopreservation on survival and function of *i*) cryopreserved fetal cardiomyocytes and *ii*) cardiomyocytes isolated from cryopreserved fetal myocardial tissues. They reported variable outcomes; for instance, long storage times, and the cell passage number decreased the cell numbers, survival, and contraction rate of cryopreserved fetal cardiomyocytes. They concluded that cryopreservation caused significant cell loss. In cardiomyocytes that were isolated from cryopreserved tissues, the mince size affected the number of contractile cardiomyocytes (the larger the tissue size, the lower the number of contractile cells). Yokomuro et al. [63] later cryopreserved human heart cells and reported low post-thaw survival (50–60% viability). They additionally reported that cryopreservation enhanced the proliferation capacity of human heart cells/tissues and reduced the immunogenicity (immune response) of heart cells. Kim et al. [64] cryopreserved human embryonic stem cell derived cardiomyocytes (hESC-CMs). They reported that the beating of cryopreserved cells was irregular and arrhythmic, and that the beating frequency was higher than that of fresh cells. They concluded that the faster beating rate was due to physiological alteration. Thus, they reported that cryopreservation triggered disruption in the nucleus, cell membrane, and mitochondria, along with physiological alterations. Preininger et al. [65] mentioned that although several studies have cryopreserved human pluripotent stem cell derived cardiomyocytes (hPSC-CMs), there is not a best strategy to effectively cryopreserve these cells that results in higher viability and maintains the cardiomyocyte characteristics with a minimum amount of alterations. They also mention that cryopreservation of hPSC-CMs could disrupt the cell–cell and cell–matrix adhesion and activate the generation of reactive oxygen species (ROS); as a result, improving the post-thaw viability of these cells seems crucial to reduce the altered functionality. Recently van den Brink et al. [66] and Zhang et al. [67] have compared the functionality of fresh and cryopreserved human induced pluripotent stem cell-derived cardiomyocytes (hiPSC-CMs) and reported different conclusions. Van den Brink et al. [66] reported viability of 95–98% based on trypan blue assay and that the replating efficiency of cryopreserved cells was lower than that for fresh cells; however, they adjusted for this by different seeding densities for fresh and cryopreserved cells. On the other hand, Zhang et al. [67] reported 60% viability based on calcein and ethidium homodimer-1 (EthD-1) assay. They observed that cryopreserved cells showed enhanced proliferation capacity (which was in agreement with the result reported by Yokomuro et al. [63]) and concluded that cryopreserved cells were less mature compared to fresh cells, which was contrary to what van den Brink et al. [66] reported. Zhang et al. [67] concluded that cryopreserved cells may have different electrophysiological properties and that cryopreserved hiPSC-CMs do not always show similar molecular and physiological properties to fresh ones and some altered responses to drugs should be taken into consideration. Both references [66,67] asserted that one systematic cryopreservation protocol, which can result in high viability, and retain the cell functionality after thaw is mandatory. With these inconsistent results, the importance of further cryopreservation studies seems even more crucial. Cardiomyocytes have been cryopreserved in different freezing media so far, which mostly contained 10–15% Me_2_SO as a cryoprotectant. There are prominent gaps in cardiomyocyte cryopreservation studies. Some of these gaps are: *i*) other types of cryoprotectants have not yet been tried (e.g., non-permeating cryoprotectants, other permeating cryoprotectants); *ii*) the quantity of Me_2_SO has not been optimized; *iii*) the procedures of cryoprotectant addition and removal have not been systematically varied (e.g., cryoprotectant–cell contact time and temperature, single or serial cryoprotectant removal); *iv*) extracellular ice nucleation has not been controlled (ice nucleating method and ice nucleating temperature); and *v*) cooling rate and cooling profile have not been optimized (e.g., plunge temperature—the temperature at which slow cooling should be stopped and cells plunged into liquid nitrogen).

A myoblast cell line derived from embryonic rat ventricular tissue (H9c2(2–1)) has been chosen as an *in-vitro* cardiac-like cell model for this study. This cell line is commercially available (ATCC® CRL1446™, Manassas, USA) and exhibits many of the properties of skeletal and cardiac muscles and is used widely as an *in-vitro* model for skeletal and cardiac muscle because of its morphological, biochemical, and electrophysiological characteristics [68–70]. Moreover, because primary cardiomyocytes are rather fragile and isolating cardiomyocytes from neonatal rat is time-consuming, and differentiating cardiomyocytes from pluripotent stem cells does not result in a defined cell type (characteristics depend on many factors, which are unknown), H9c2 has been chosen for this study to ensure reproducible results. The H9c2 manufacturer has recommended complete growth medium plus 5% Me_2_SO as the cryopreservation solution for this cell type [71]. Hence, we first cryopreserved H9c2 in its complete growth medium plus 5% (w/w) Me_2_SO; then we investigated the combination of 5% (w/w) Me_2_SO + 6% (w/w) HES in complete growth medium because as mentioned earlier, this combination has been shown to be suitable for many other cell types [26,33,72–77]. We chose to incubate cells with either 5% Me_2_SO or 5% Me_2_SO + 6% HES for 15 minutes at 0°C to reduce the contact time and temperature to get better results when Me_2_SO is used, and it has been shown to be the best incubation time and temperature by previous studies [26,76]. Finally, we investigated glycerol, which has been shown to be effective for other cell types [18,23,58] as a cryoprotectant for H9c2 in order to find a Me_2_SO-free cryoprotectant for H9c2 for use in cases when using Me_2_SO is undesired in a particular application. We investigated *i*) 5% (w/w) glycerol with one hour incubation at room temperature; *ii*) 10% (w/w) glycerol with one hour incubation at room temperature; and *iii*) 5% (w/w) glycerol with two hours incubation at room temperature. As mentioned earlier here, glycerol needs more time and higher temperature to penetrate the cells and provide protection against cooling injuries; hence, we started with 5% glycerol for one hour at room temperature because it was mentioned as the best incubation time and temperature for swine colostrum-derived cells and human cerebral microvascular endothelial cells [58,59]. Afterwards, we changed the glycerol concentration and incubation time to get better results specifically for H9c2 cells.

In addition to investigating different types of cryoprotectants, different cryoprotectant concentrations, different incubation times and temperatures, another parameter that is important in a successful cryopreservation protocol is the temperature at which extracellular ice nucleation is induced. It has been shown that intracellular ice formation—during plunging cells into liquid nitrogen—depends on the temperature at which the extracellular ice is nucleated; extracellular ice nucleation at the highest practical temperature reduces the intracellular ice formation that happens upon plunge into liquid nitrogen when using lower ice nucleation temperatures [78]. Ice nucleation at −5°C was chosen in this study.

Other important factors affecting successful cryopreservation: the cooling profile (specifically the plunge temperature) and the best method for cryoprotectant removal have been investigated in this study as well.

The H9c2 cell line was derived from embryonic rat heart tissue and consists of mononucleated myoblasts (flat spindle shape) with the morphology changing to multinucleated myotubes (large rounded/polygonal shape) upon fusion. Fusion is a very slow process that takes several weeks to occur and only 85% of mononucleated myoblasts change to multinucleated myotubes. Decreasing the concentration of serum in culture medium (from 10% to 1%) expedites the fusion to a shorter time with over 95% of mononucleated myoblasts changing to multinucleated myotubes [69]. Because H9c2 myoblasts have the ability to be differentiated into either skeletal or cardiac myotubes (myocytes) [79–81], decreasing the serum concentration only favors the skeletal fusion [69,82]. However, when all-trans retinoic acid (ATRA) is added to the culture medium (in addition to serum concentration reduction), H9c2 cells differentiate into a cardiac phenotype [83,84] and they are positive for expression of cardiac markers [70,82,85,86]. It is known that heart muscle cells have far more mitochondria than any other cells because these cells contract (beat) and need much more energy than other cells [87]. Therefore, it is expected to observe *i*) a larger mass of mitochondria as well as *ii*) a change in morphology and structure when H9c2 mononucleated myoblasts differentiate to cardiac multinucleated myotubes. Troponin is a protein that regulates calcium for the contractile function in cardiac and skeletal muscle cells [88,89]. Troponin I is a more specific marker for myocardium compared to troponin T. Cardiac myotubes (myocytes) express both troponin T and troponin I; though, the specificity is in the point that cardiac troponin T is also expressed in skeletal myotubes whereas cardiac troponin I is expressed in cardiac myotubes only [90]. For this reason, troponin I was chosen as a cardiac marker in this study to observe if H9c2 myotubes express troponin I as they differentiate to cardiac myotubes after serum reduction and ATRA stimulation.

We report a systematic study of H9c2 cell cryopreservation by slow cooling in the presence of extracellular ice, which investigates important parameters affecting the post-thaw cell survival and functionality with the aim of reporting a final optimized cryopreservation protocol to cryopreserve this cell type. The knowledge gained will be useful in designing protocols for cryopreservation of other types of heart cells and cardiac biologics such as engineered tissues.

## Materials and methods

### Cell cultures

H9c2(2–1) (ATCC CRL-1446) cells were received on dry ice cryopreserved in complete growth medium, which was comprised of Dulbecco’s Modified Eagle Medium (DMEM; ATCC 30–2002) plus 10% v/v FBS (GIBCO 12483020, Thermo Fisher Scientific) supplemented with 5% (v/v) Me_2_SO and were kept in a liquid nitrogen storage dewar until use. The H9c2 vial contained 1 mL of cells in suspension that was rapidly thawed in a 37°C water bath. To remove the Me_2_SO, the thawed 1 mL of cell suspension was added to 9 mL of complete growth media (i.e., DMEM base medium, plus 10% (v/v) FBS) and centrifuged at 140 g for 5 minutes and the supernatant removed. The cell pellet was then resuspended and cultured in complete media (i.e., DMEM base medium, plus 10% (v/v) FBS) in 75-cm^2^ tissue culture flasks (T-flask) (Corning T75, U-shaped canted neck cell culture flask with vent cap, Millipore-Sigma) in a humidified incubator (Eppendorf, CellXpert C170, Hamburg, Germany) at 37°C and 5% CO_2_. The growth medium was changed every 2–3 days until the cells reached 60–85% surface coverage (see S1–S3 Figs in S1 File) as observed using a Labovert phase contrast microscope (Leitz, Los Angeles, CA, USA). Then, sub-culturing/passaging was carried out using 0.25% (w/v) trypsin-0.53 mM EDTA detachment solution (GIBCO 25200072, Thermo Fisher Scientific). First, the growth medium was vacuum aspirated; then, the cell monolayer was washed with 2–3 mL trypsin/EDTA (and vacuum aspirated); next, 3 mL of trypsin/EDTA was added, and the culture flask was incubated at 37°C for 3 minutes. 6 mL of H9c2 complete growth media (double the volume of trypsin/EDTA) was added into the flask to stop trypsinization, and cell suspension was collected in a 50-mL centrifuge tube. Another 6 mL of complete growth medium was added to wash the flask and collect the cell residuals. The collected cell suspension was centrifuged at 140 g for 5 minutes at room temperature (Eppendorf 5810R, Mississauga, Canada). The supernatant was vacuum aspirated, and the cell pellet was resuspended in complete growth medium. 100 μL of cell suspension was diluted in 10 mL of Isoton II (8546719, Beckman Coulter, Inc., Mississauga, Canada) to measure the cell concentration using a Z2 Coulter particle count and size analyzer (Beckman Coulter). The rest of the cell suspension (in complete growth medium) was either used for the same day experiments or reseeded for future experiments. Those cells that were to be used for the same day experiments were stored at 0°C (to avoid cell clumping) for a maximum of 10 minutes before use. Cells from passage numbers 2–10 (see S4–S6 Figs in S1 File) were used for these experiments at concentrations from 800,000 to 1,500,000 cells per millilitre.

### Graded freezing without cryoprotectants

H9c2 cells suspended in complete growth medium were subjected to interrupted slow cooling (graded freezing) in the absence of cryoprotectants. Aliquots of 200 μL of H9c2 cell suspension were transferred to 6×50 mm borosilicate glass culture tubes (Kimble 73500–650, Thermo Fisher Scientific, Ottawa, Canada). Borosilicate glass culture tubes were then corked and transferred into a programmable stirring methanol bath (FTS Systems, Stone Ridge, USA) and were cooled at 1°C/min. The bath temperature was measured by a T-type thermocouple and OMEGA USB Data Acquisition System (OMB-DAQ-55, Spectris Canada, St-Eustache, QC). Three thermocouples were first placed in the water portion of an ice–water bath for temperature referencing at 0°C, and the temperature of each thermocouple was recorded. The temperature of the methanol bath was set at −5°C, and one thermocouple was placed directly in the methanol bath; another thermocouple was inserted in a borosilicate glass culture tube containing 200 μL of growth media (to perform a temperature measurement representative of H9c2 suspensions), and the last thermocouple was kept in ice water. Samples in the methanol bath were allowed to equilibrate at −5°C for 2 minutes. Extracellular ice was then nucleated by quickly touching the glass tubes with forceps cooled in liquid nitrogen (Praxair Canada Inc., Edmonton, Canada), and samples were left for 3 minutes at −5°C to release the latent heat of fusion. The freezing point of the H9c2 suspension with permeating cryoprotectant (Me_2_SO) was calculated according to Eq 2 noted in [91] (or Eq 3 noted in [92]), as well as Eq 3 noted in [76] and found to be −1.3°C. It has been recommended that nucleation is best performed at 3°C below the solution freezing point [76]. It has been shown in previous studies that −5°C was a suitable temperature to induce extracellular ice nucleation [59,77,93,94]. When cells were placed in the methanol bath (at −5°C) they were still unfrozen. This phenomenon is due to supercooling [95]. It is not preferable that the intracellular solution be significantly supercooled because supercooling increases the chance of intracellular ice formation. Extracellular ice nucleation at a reasonably high sub-zero temperature is the best way to relieve the supercooling and can either be induced [96] or occur spontaneously. Purposeful extracellular ice nucleation at a specific temperature (−5°C) was chosen to prevent ice nucleation at random temperatures which would increase the variability of results. Then, the methanol bath was set to a cooling rate of 1°C/min. At intermediate temperatures of −10, −20, −30, and −40°C, the temperature data of all thermocouples were recorded, and two sample tubes were rapidly thawed in a 37°C water bath (Sheldon 382 Manufacturing Inc., Cornelius, USA) until all ice was melted (direct-thaw samples), and two other sample tubes were plunged into liquid nitrogen; the samples were kept in liquid nitrogen for at least half an hour, and then thawed rapidly in the 37°C water bath (plunge-thaw samples). After thawing, cells were immediately stained for membrane integrity assessment at room temperature.

### Cryoprotectants

Three different cryoprotectant solutions were used in this study: *i*) permeating cryoprotectant: 5% w/w Me_2_SO (0.65 M, D128, Fisher Scientific, Ottawa, Canada) in complete growth medium; *ii*) a combination of a permeating and a non-permeating cryoprotectant: 5% (w/w) Me_2_SO + 6% (w/w) HES (low molecular weight hydroxyethyl starch; 20% pentastarch solution, 200 mg/mL, Preservation Solutions Inc. (PSI), Elkhorn, USA) in complete growth medium, and *iii*) 5% (or 10%) w/w glycerol (0.55 M, G–153, Fisher Scientific) in complete growth medium. Cryoprotectants were prepared at 2X the final w/w % concentration in complete growth medium, and then added as a single dose (all at once) to the cell suspension in complete growth medium in a 1:1 ratio by weight to give the desired final concentration of cryoprotectants.

### Graded freezing with cryoprotectants

**Fig 1:**
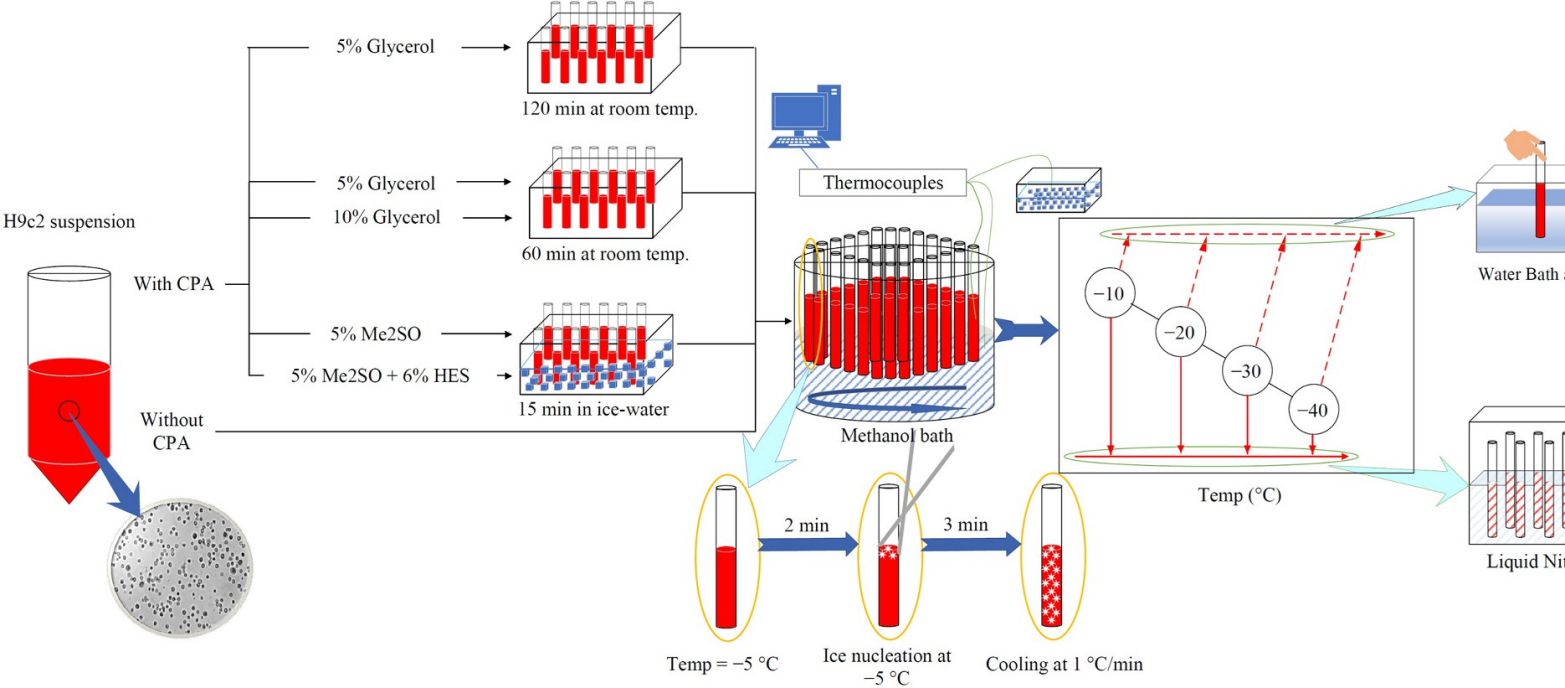
Schematic diagram of graded freezing for H9c2 cells with and without cryoprotectants. In each different experiment, H9c2 suspension was either incubated with cryoprotectants or not. The methanol bath was set at −5°C. H9c2 samples were placed inside the methanol bath and after 2 minutes of equilibration, extracellular ice nucleation was induced by touching the glass tube with pre-cooled forceps. Cells were held at −5°C for 3 minutes. Then cooling started at 1°C/min. At each sub-zero temperature, some samples were directly thawed in a water bath, while others were plunged into liquid nitrogen. After at least half an hour being in liquid nitrogen, those samples were thawed in the water bath as well.

H9c2 cells in suspension then were subjected to graded freezing in the presence of cryoprotectants described earlier. Samples with 5% (w/w) Me_2_SO and/or 5% (w/w) Me_2_SO + 6% (w/w) HES were incubated at 0°C for 15 minutes, samples with 10% w/w glycerol were incubated at room temperature for one hour, and samples with 5% (w/w) glycerol were incubated at room temperature for either one or two hours, before starting the graded freezing. Aliquots of 200 μL of H9c2 cells mixed with cryoprotectants were transferred to glass culture tubes and corked. The experimental samples with 5% (w/w) Me_2_SO and/or 5% (w/w) Me_2_SO + 6% (w/w) HES were then transferred from 0°C into the methanol bath (which was set at −5°C), while samples with 5% or 10% w/w glycerol were transferred from room temperature into the methanol bath (which was set at −5°C) and graded freezing was performed for each condition through the procedure as described in the section, “Graded freezing without cryoprotectants”. Fig 1 is a schematic diagram of all the stages of a graded freezing procedure including cryoprotectant addition, connecting thermocouples to record the temperature change, placing cells in the methanol bath, extracellular ice nucleation, holding cells for 3 minutes after ice nucleation, cooling at 1°C/min to different sub-zero temperatures, directly thawing some samples from each sub-zero temperature, and finally plunging some samples into liquid nitrogen from each sub-zero temperatures and then thawing those samples.

### Membrane integrity assessment by flow cytometry

Thawed cells were immediately analyzed with a Coulter Epics XL-MCL flow cytometer (Beckman Coulter Inc., USA) equipped with a 488 nm laser using a dual fluorescent stain composed of SYTO 13 (Green Fluorescent Nucleic Acid Stain, Thermo Fisher Scientific, USA) and GelRed (Nucleic Acid Gel Stain, Biotium, USA). The stain mixture was prepared by mixing 12 μL of SYTO 13 (5 mM Solution in Me_2_SO), 19 μL GelRed (10,000X in water), and 220 μL distilled water in a microtube (228 μM SYTO 13 and 720X GelRed) and was kept at 0°C in the dark until use [26]. Two cell samples in glass culture tubes (each glass tube contained 200 μL, so the total amount of cell solution was 400 μL) were transferred into a flow cytometry tube (T405-2 Simport Scientific Inc., 12mm × 75mm); because the required sample size for the flow cytometry was 400 μL. Then, 20 μL of dye solution was added, and incubated at room temperature for 10 min in the dark [94]. Then the flow cytometry tube was vortexed and placed in the flow cytometer for membrane integrity assessment. The flow cytometer settings used are described elsewhere [76,94]. The data were analyzed with flow cytometry analysis software (KaluzaTM v1.2 from Beckman-Coulter). The membrane integrity (MI) was calculated by dividing the total number of live cells (membrane-intact cells with green fluorescence) by the total number of cells (membrane-intact cells with green fluorescence, membrane-damaged cells with red fluorescence, as well as doubly stained cells), which is shown by Eq 1.


$$\%\;\text{MI}=\frac{\text{High}\;\text{green}\;\text{events}}{\text{High}\;\text{green}\;\text{events}+\text{High}\;\text{red}\;\text{events}+\text{doubly-stained}\;\text{events}\;}\times 100\text{\%}$$ (1)

### Membrane integrity assessment by trypan blue exclusion method

Twenty μL of thawed cells were transferred into a tube and were well-mixed with 20 μL of trypan blue (0.4%, BioWhittaker Lonza, Walkersville, USA) and then left at room temperature for 3 minutes [97]. About 10 μL of the stained cells in suspension were delivered to each side of a hemacytometer at the edge of the coverslip, and the hemacytometer was placed on the stage of a bright-field microscope (Diavert, Wild Leitz, Ottawa, Canada) to count the viable (clear cells) and non-viable cells (blue-colored cells). Viable cells as well as non-viable cells were counted for each side of the hemacytometer. The membrane integrity percentage was calculated by Eq 2.


$$\%\;\text{MI}=\frac{\text{TB-excluding}\;\text{cells}\;\text{counted}\;\text{for}\;\text{side}\;1+\text{TB-excluding}\;\text{cells}\;\text{counted}\;\text{for}\;\text{side}\;2}{\text{all}\;\text{cells}\;\text{for}\;\text{side}\;1+\text{all}\;\text{cells}\;\text{for}\;\text{side}\;2}\times 100\%$$ (2)

### Cryoprotectant removal after thaw

**Fig 2:**
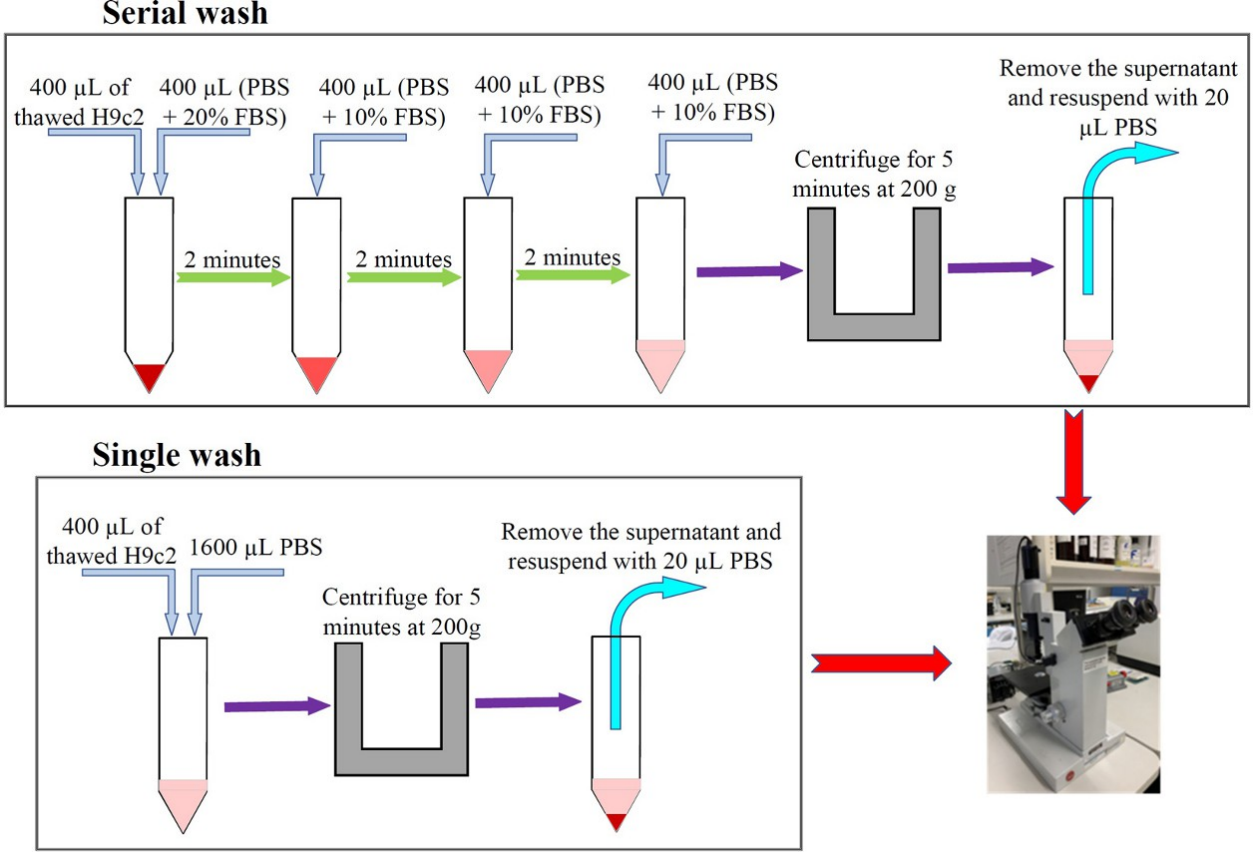
Cryoprotectant removal procedures for serial and single wash. H9c2 suspensions were subjected to 5% Me_2_SO + 6% HES or 5% glycerol (two-hour incubation) before being cooled at 1°C/min to ˗40°C and transferred into liquid nitrogen. After at least half an hour being kept in liquid nitrogen, cells were then thawed rapidly in a water bath (37°C). Cryoprotectants were then removed by either adding PBS at 4X the volume of cell suspension before centrifuging the cells (single wash) or adding PBS + FBS at 1X the volume of cell suspension 3 times and waiting for 2 minutes after each addition before centrifuging the cells (serial wash). Supernatant was then removed, and cells were resuspended in PBS for membrane integrity assessment with trypan blue.

Post-thaw samples were subjected to membrane integrity assessment without cryoprotectant removal as well as after cryoprotectant removal. For those experiments for which cryoprotectant removal was not performed (i.e., optimization experiments for which immediate post-thaw membrane integrity could determine the cell response to different experimental conditions) the immediate post-thaw membrane integrity was measured by flow cytometry; however, for those experiments for which cryoprotectant removal was necessary after thaw (i.e., assessment of functionality after the optimal cryopreservation protocols had been determined), the membrane integrity was measured by trypan blue exclusion to minimize the sample required (twenty μL needed for trypan blue compared to 400 μL for flow cytometry; after cryoprotectant removal, samples needed to be re-cultured for functional assessment, as a result, having enough sample for seeding was important). Cryoprotectant removal was performed through *i*) single dilution and *ii*) serial dilution (Fig 2). For cryoprotectant removal from H9c2 suspension by single wash, the contents of a single glass culture tube (200 μL) were transferred into a 15-mL centrifuge tube, and phosphate-buffered saline (PBS, Life Technologies, Grand Island, USA) was added at 4X the volume of cell suspension (800 μL); cells were then centrifuged at 200 g for 5 minutes, supernatant was removed, and the cells re-suspended in 20 μL PBS before staining with trypan blue. For serial wash, after the contents of the glass culture tube were transferred into a 15-mL centrifuge tube, 200 μL PBS supplemented with 20% (v/v) FBS was added, and cells were incubated for 2 minutes at room temperature, then 200 μL PBS supplemented with 10% (v/v) FBS was added, and cells were incubated for another 2 minutes at room temperature, this step was repeated 2 more times, then cells were centrifuged at 200 g for 5 minutes, the supernatant was removed, and the cells re-suspended in 20 μL PBS before staining with trypan blue. To optimize the cryoprotectant removal method, the membrane integrity was measured with trypan blue either immediately after thaw or after single wash as well as after serial wash to compare the results and choose the best method for cryoprotectant removal.

### H9c2 myoblast differentiation to myotubes

Fresh and post-thaw cells (after cryoprotectant removal) were seeded on fibronectin-coated (Fibronectin bovine plasma, Sigma-Aldrich F1141-2MG) glass coverslips (VWR 89015–725, 12 mm diameter) in 24-well plates (Thermo Scientific 142475, Denmark) at a density of 5000 cells/cm^2^ (10,000 cells per well). Fresh cells were cultured in complete growth medium, and after-thaw samples were cultured in complete growth medium supplemented with penicillin/streptomycin (100X, Cytiva HyClone™ SV30010) to prevent any contamination during monolayer formation. Both fresh and post-thaw samples were incubated in a humidified incubator at 37°C and 5% CO_2_ overnight. The next day, the serum was deprived from 10% to 1% (v/v) in culture medium, in addition to daily all-trans retinoic acid (ATRA) (72264, Stem Cell Technologies, Vancouver, Canada) stimulation. ATRA arrived as a yellow powder that was stable at −20°C and was protected from light. The manufacturer recommended using Me_2_SO as the solvent for this compound due to its low solubility in aqueous media [98]. Around 30 mg of ATRA was dissolved in 10 mL Me_2_SO, sterile-filtered (LifeGene, SF0.45PES, 0.45 μm) in the biosafety cabinet under subdued light, aliquoted (100 μL/tube) into microcentrifuge tubes (10 mM), and stored at –20°C. On the day of stimulation, 10 μL of 10 mM ATRA solution was added to 0.99 mL growth medium with 1% FBS (ATRA concentration: 10^−4^ M in 1% Me_2_SO). Then, 10 μL of 10^−4^ M solution was added to another 0.99 mL of the same medium (concentration: 1 μM ATRA in 0.01% Me_2_SO). Five μL of 1 μM ATRA was added to 500 μL of growth medium with 1% FBS in each well, so that the final ATRA concentration was 10 nM in 0.0001% Me_2_SO). The important note regarding ATRA stimulation was the final Me_2_SO concentration in cell culture, which should not be above 0.1% due to potential cell toxicity. The medium (serum deprived medium) was replaced every 2 days, and ATRA was added every day. After 3 and 7 days under differentiation conditions, the monolayers were observed under a phase contrast microscope to see the multinucleated myotube formation and were ready for functional assessment.

### Mitochondrial mass assay using the mitochondrial probe Mito Tracker Red

Mito Tracker Red is a red-fluorescent dye that stains functional mitochondria [99]. Cardiac myocytes have numerous mitochondria, to provide sufficient energy for their continuous activity [100]. Thus, the increase of mitochondrial mass during differentiation of H9c2 myoblasts into cardiac myotubes is used here as an assessment to observe the differentiation as well as a comparison between fresh and cryopreserved H9c2 functionalities. Mito Tracker Red (CMXRos, Invitrogen M7512, Oregon, USA) comes as lyophilized solid in vials labeled 50 micrograms per vial, and is stored at –20°C, and protected from light. Ninety-four μL of anhydrous Me_2_SO was added to a vial of Mito Tracker Red to prepare a 1 mM stock solution [101]. After 3 and 7 days under differentiation conditions, 10 μL of 1 mM solution was added to 0.99 mL PBS (1X) (Mito Tracker Red concentration: 10^−5^ M in 1% Me_2_SO); then 25 μL of 10^−5^ M Mito Tracker Red was added to 500 μL of medium with 1% FBS in each well of the 24-well plate (final Mito Tracker Red concentration: 500 nM in 0.01% Me_2_SO). The 24-well plate was then transferred into the incubator for 30 minutes, then it was ready for imaging with a fluorescent microscope (Leitz, Dialux 22).

### Immunocytochemistry

Fresh and post-thaw cells were assessed for troponin I expression after differentiation to cardiac myotubes. After 3 and 7 days under differentiation-inducing conditions, fresh and thawed H9c2 monolayers (in 24-well plates) were fixed with 3.7% paraformaldehyde (Sigma-Aldrich, F8775) in PBS (500 μL at each well) for 15 minutes at room temperature. Then, the paraformaldehyde was removed and the wells were rinsed 3X with 500 μL PBS. Washing with PBS was performed for 5 minutes each time using a mini shaker (Fisher Scientific Nutating Mixer, 05-450-213, speed setting = 24) in the fridge, based on the manufacturer’s recommendation [102]. After washing, 500 μL permeabilization buffer (0.2% Triton X-100 in PBS, Fisher Scientific, BP151) was added in each well, and incubated for 5 minutes at room temperature, followed by washing with PBS and blocking with 1% goat serum (Life Technologies PCN 5000) in PBS for at least 60 minutes at room temperature in the dark. The primary antibody (mouse monoclonal [4C2] to cardiac troponin I, Abcam, ab10231) was diluted in PBS containing 1% goat serum (1:200) and was added (300 μL in each well), and incubated first at room temperature for 2 hours, and then overnight at 4°C in the dark. The next day, after washing off the primary antibody, the secondary antibody (goat anti-mouse IgG (FITC), Abcam 6785) was diluted in PBS containing 1% goat serum (1:200), added to each well (300 μL) and incubated for 3 hours at room temperature in the dark. The antibody solution was then removed and the coverslips were washed 3X with PBS. Then, coverslips were removed from the plate, and transferred cell side facing down on a microscope slide and imaged using the fluorescent microscope. It should be noted here that all the incubation times and temperatures in this procedure have been optimized after performing all the scenarios given by the manufacturer. Incubation times and temperatures for each step are those that provided the best results.

### Statistical analysis

Graded freezing experiments were performed independently at least 3 times, and the means and standard deviations were calculated using Microsoft Excel 2019. Statistical analysis of differences was calculated using paired, 2-sample t-tests. Differences between means of data sets were considered statistically significant if p values were ≤ 0.05.

## Results

### Optimization of cryopreservation of H9c2 in suspension

**Fig 3:**
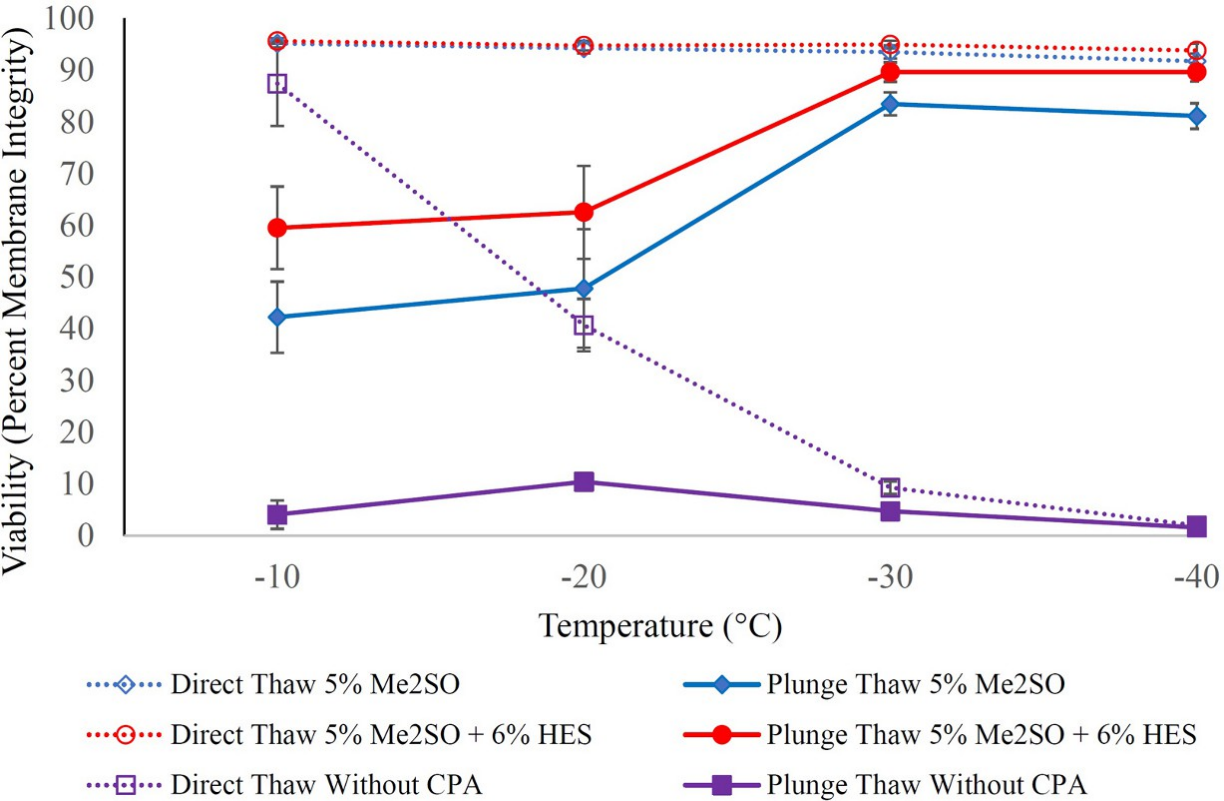
The response of H9c2 suspension to graded freezing with and without Me_2_SO-containing cryoprotectants. H9c2 suspension was cooled at 1°C/min to different sub-zero temperatures either without cryoprotectant agent (CPA) or in the presence of 5% Me_2_SO or 5% Me_2_SO + 6% HES. Samples were either directly thawed from sub-zero temperatures (open symbols, dashed lines) or were first plunged into liquid nitrogen before being thawed at 37°C (solid symbols and lines). Cells were then subjected to membrane integrity assessment based on SYTO 13/GelRed. Data points are the mean of independent experiments (at least 3 repeats) and error bars indicate standard deviation.

**Fig 4:**
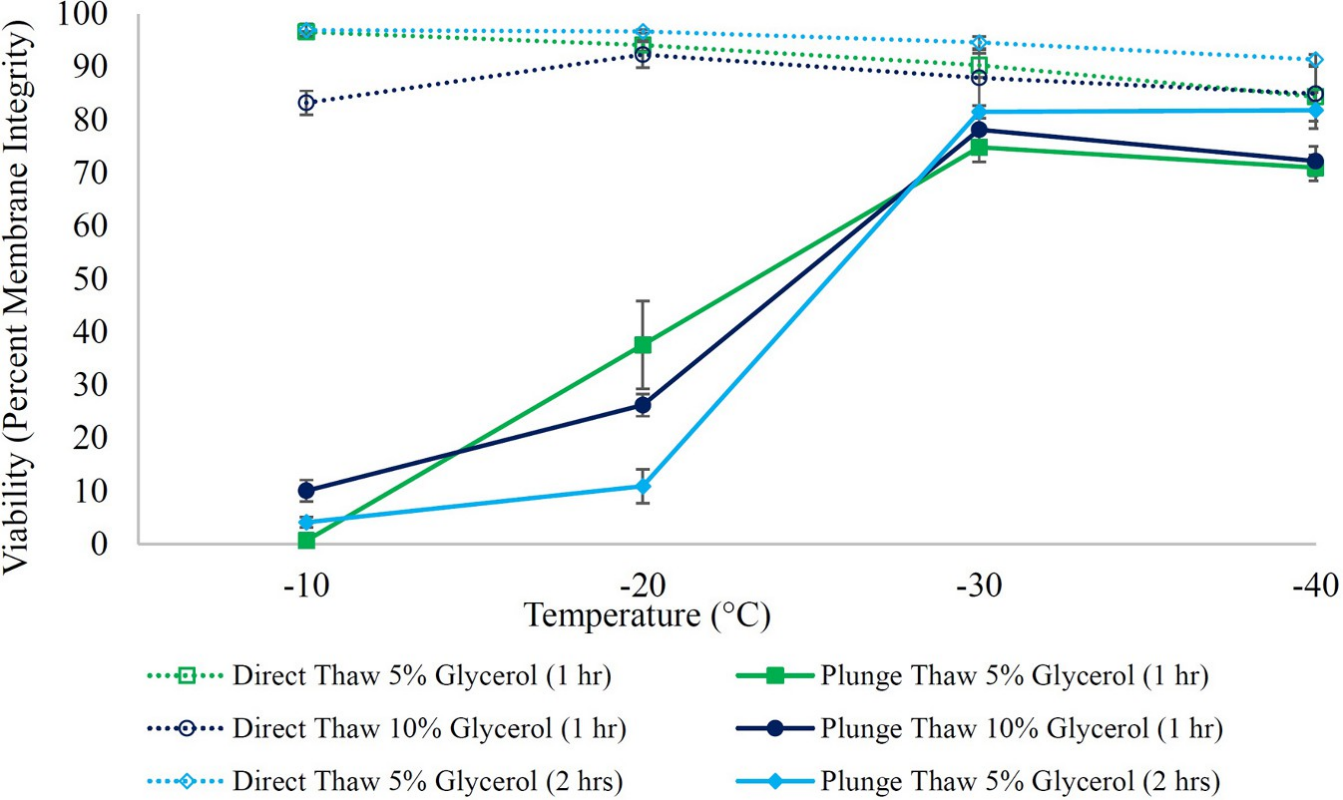
H9c2 response to graded freezing in the presence of 5% glycerol either incubated for one or two hours as well as 10% glycerol incubated for one hour at room temperature. H9c2 suspension was incubated with 5% glycerol (either for one or two hours) and 10% glycerol (for one hour) at room temperature before being cooled at 1°C/min to different sub-zero temperatures. Samples were either directly thawed from sub-zero temperatures (open symbols, dashed lines) or were first plunged into liquid nitrogen before being thawed at 37°C (solid symbols and lines). Cells were then subjected to membrane integrity assessment based on SYTO 13/GelRed. Data points are the mean of independent experiments (at least 3 repeats) and error bars indicate standard deviation.

Graded freezing is an experimental tool to facilitate the understanding of cellular responses during freeze/thaw processes to distinguish cooling injuries that occurred during the slow cooling to the intermediate temperatures (direct-thaw samples) from those that happened during the rapid cooling from each intermediate temperature to the liquid nitrogen storage temperature (plunge-thaw samples) [103–105]. The cell response is depicted in Figs 3 and 4.

In Fig 3, dashed lines represent the viability (membrane integrity by SYTO 13/GelRed) of samples that were directly thawed from the intermediate temperatures (direct-thaw; indicating slow-cool injury accumulated on the way to the intermediate temperature). Solid lines represent the membrane integrity of H9c2 cells that were plunged into liquid nitrogen from the intermediate temperature, stored for at least half an hour, and then thawed (plunge-thaw; the difference between direct-thaw and plunge thaw results indicates the further rapid-cool injury that occurs upon plunge into liquid nitrogen from the intermediate temperature). The cooling rate for cells when they were plunged into liquid nitrogen from −40°C was 1041 ± 129°C/min (see S7 Fig in S1 File). Similarly, the warming rates for cells when they were directly thawed from −40°C (direct-thaw), as well as when they were thawed after plunging into liquid nitrogen where they remained for at least half an hour (plunge-thaw) were 223 ± 32°C/min (see S8 Fig in S1 File) and 1398 ± 300°C/min (see S9 Fig in S1 File), respectively.

Purple lines (Fig 3) show the response of H9c2 cells in the absence of cryoprotectants. The low membrane integrities of the purple dashed and solid lines clearly indicate that the slow cooling injuries and rapid cooling injuries, respectively, are not mitigated. Cells without cryoprotectant have already experienced slow cooling injuries when directly thawed from −10°C (membrane integrity ~76%). As cooling continues (to lower sub-zero temperatures) slow cooling injuries increase and are manifested in membrane integrity reduction with decreasing temperature of direct-thaw samples (reaching to ~1% at −40°C). Plunge-thaw samples show very low viability after having been plunged from −10°C, likely because there was enough intracellular water at −10°C that could freeze during plunging into liquid nitrogen causing cell damage by intracellular ice formation. By losing sufficient intracellular water by slow cooling to −20°C, the plunge-thaw membrane integrity increases slightly (reaching ~10%); however, plunge-thaw membrane integrity reduces to ~1% at plunge temperatures of −30°C and −40°C because cells were already killed by slow cooling injuries. 5% Me_2_SO as well as 5% Me_2_SO + 6% HES (blue and red dashed lines) could mitigate the slow cooling injuries (direct-thaw viabilities around 90% during cooling down to −40°C). Cells with permeating cryoprotectants can tolerate lower sub-zero temperatures because of freezing point depression and have reduced slow-cool injury due to less ice formed and thus less solute concentration at a given temperature; hence, cells can be slowly cooled for long enough to lose enough water to minimize the intracellular ice formation during plunge from those low sub-zero temperatures into liquid nitrogen. For this reason, plunge-thaw viabilities (solid lines) increased when using 5% Me_2_SO (from 42.8 ± 6.9% at −10°C to 81.1 ± 2.5% at −40°C) as well as 5% Me_2_SO + 6% HES (from 63.1 ± 7.4% at −10°C to 90.9 ± 2.1% at −40°C). Post-thaw viabilities were significantly higher for 5% Me_2_SO + 6% HES at each sub-zero temperature compared to when 5% Me_2_SO was used alone because of the presence of HES (p values for each temperature are shown in Table 1). The highest survival achieved for 5% Me_2_SO + 6% HES was either 90.8 ± 2.2% at −30°C or 90.9 ± 2.1% at −40°C (p = 0.4). These values were significantly better than the maximum membrane integrities achieved for 5% Me_2_SO of either 83.5 ± 2.2% at −30°C or 83.1.1 ± 2.5% at −40°C, respectively (Table 1).

**Table 1:** p values for membrane integrities at different experimental conditions comparing 5% Me_2_SO + 6% HES and 5% Me_2_SO alone.

Experimental condition	5% Me_2_SO + 6% HES % Membrane Integrity ± St. Dev.	5% Me_2_SO % Membrane Integrity ± St. Dev.	p value
Direct thaw, −10°C	96.6 ± 1.1	95.2 ± 1.3	0.03
Direct thaw, −20°C	96.0 ± 1.6	94.2 ± 1.8	0.03
Direct thaw, −30°C	96.3 ± 1.3	93.5 ± 2.2	0.007
Direct thaw, −40°C	95.5 ± 1.1	91.8 ± 2.5	0.003
Plunge thaw, −10°C	63.1 ± 7.4	42.8 ± 6.9	0.002
Plunge thaw, −20°C	67.7 ± 7.1	47.8 ± 11.5	0.01
Plunge thaw, −30°C	90.8 ± 2.2	83.5 ± 2.2	0.0007
Plunge thaw, −40°C	90.9 ± 2.1	81.1 ± 2.5	0.0001

To explore a Me_2_SO-free cryopreservation protocol for H9c2 in suspension, we first started with 5% (w/w) glycerol and incubated it with cells for one hour at room temperature before starting the slow cooling (Fig 4). Green dashed and solid lines indicate direct-thaw and plunge-thaw viabilities, respectively, for 5% glycerol with one hour incubation. The membrane integrity for direct-thaw samples decreased gradually from 96.6 ± 0.1% at −10°C to 84.4 ± 0.8% at –40°C in the presence of 5% glycerol that had been incubated for 1 hour. Next, the effect of glycerol concentration was investigated by increasing the glycerol concentration to 10% (w/w). Dark blue lines (Fig 4) show the viability for 10% glycerol with one hour incubation time. Table 2 shows p values for post-thaw viabilities to compare 5% and 10% glycerol when incubated at room temperature for one hour. It can be noticed that increasing the concentration of glycerol did not significantly improve the direct-thaw viabilities.

**Table 2:** p values for membrane integrities at different experimental conditions comparing post-thaw viabilities for 5% and 10% glycerol when incubated at room temperature for one hour.

Experimental condition	5% glycerol % Membrane Integrity ± St. Dev.	10% glycerol % Membrane Integrity ± St. Dev.	p value
Direct thaw, −10°C	96.6 ± 0.1	83.2 ± 2.2	0.007
Direct thaw, −20°C	94.1 ± 1.1	92.3 ± 2.4	0.1
Direct thaw, −30°C	90.3 ± 2.3	87.9 ± 5.2	0.2
Direct thaw, −40°C	84.4 ± 0.8	84.9 ± 5.2	0.4
Plunge thaw, −10°C	0.7 ± 0.5	10.1 ± 2.0	0.007
Plunge thaw, −20°C	37.6 ± 8.3	26.2 ± 2.1	0.07
Plunge thaw, −30°C	74.9 ± 2.7	78.2 ± 3.2	0.2
Plunge thaw, −40°C	71.0 ± 2.4	72.2 ± 2.8	0.002

To investigate the role of incubation time, cells were then incubated for two hours (at room temperature) in 5% glycerol. Sky blue lines in Fig 4 show the membrane integrity for 5% glycerol with two hours incubation. p value at −40°C in Table 3 indicates that direct-thaw viability improved significantly when incubation time increased from one hour to two hours for 5% glycerol at room temperature. p values at −30 and −40°C in Table 3 indicate that increasing the incubation time also improved the plunge-thaw viabilities for 5% glycerol.

**Table 3:** p values for membrane integrities at different experimental conditions comparing post-thaw viabilities for 5% glycerol when incubated at room temperature for one and two hours.

Experimental condition	5% glycerol (one-hour incubation) % Membrane Integrity ± St. Dev.	5% glycerol (two-hour incubation) % Membrane Integrity ± St. Dev.	p value
Direct thaw, −10°C	96.6 ± 0.1	96.9 ± 1.1	0.4
Direct thaw, −20°C	94.1 ± 1.1	96.7 ± 0.3	0.02
Direct thaw, −30°C	90.3 ± 2.3	94.6 ± 1.2	0.09
Direct thaw, −40°C	84.4 ± 0.8	91.4 ± 0.9	0.007
Plunge thaw, −10°C	0.7 ± 0.5	4.1 ± 1.0	0.04
Plunge thaw, −20°C	37.6 ± 8.3	10.9 ± 3.2	0.03
Plunge thaw, −30°C	74.9 ± 2.7	81.5 ± 1.2	0.04
Plunge thaw, −40°C	71.0 ± 2.4	81.8 ± 3.5	0.05

Interestingly, Fig 4 shows that the viability for the −20°C plunge-thaw samples was higher when glycerol was incubated for one hour than when glycerol was incubated for two hours. This same observation was not found in a similar study with a different cell type [59] indicating the complexity of the action of glycerol which has a permeation-dependent behavior with cell membrane permeability being cell-type dependent. Though not fully understood, this behavior at a non-optimal plunge temperature is not that important for cryopreservation protocol development which aims to use appropriate plunge temperatures.

The maximum membrane integrity for 5% glycerol (one-hour incubation) was 74.9 ± 2.9% when cells were plunged into liquid nitrogen at −30°C; the highest membrane integrity for 10% glycerol (one-hour incubation) was 78.2 ± 3.2% when cells were plunged into liquid nitrogen at −30°C; and the highest value for membrane integrity for 5% glycerol (two-hour incubation) was either 81.5 ± 1.2% when cells were plunged into liquid nitrogen at −30°C or 81.8 ± 3.5% when cells were plunged into liquid nitrogen at −40°C (p = 0.5). The highest plunge-thaw membrane integrities for 5% glycerol (one-hour incubation) and 10% glycerol (one-hour incubation) that both were at plunge temperature of −30°C were comparable, p = 0.2 (Table 2). However, the highest membrane integrities for 5% glycerol (one-hour incubation), which was at −30°C, and for 5% glycerol (two-hour incubation), which was at either −30°C or −40°C, were different (p = 0.04) (Table 3). 5% glycerol with two hours incubation at room temperature (plunge temperature of −40°C) was chosen for further functional assessments because the direct-thaw viabilities at −40°C as well as the plunge-thaw viabilities at −30 and −40°C (Table 3) were significantly higher for the two-hour incubation compared to one-hour incubation.

**Fig 5:**
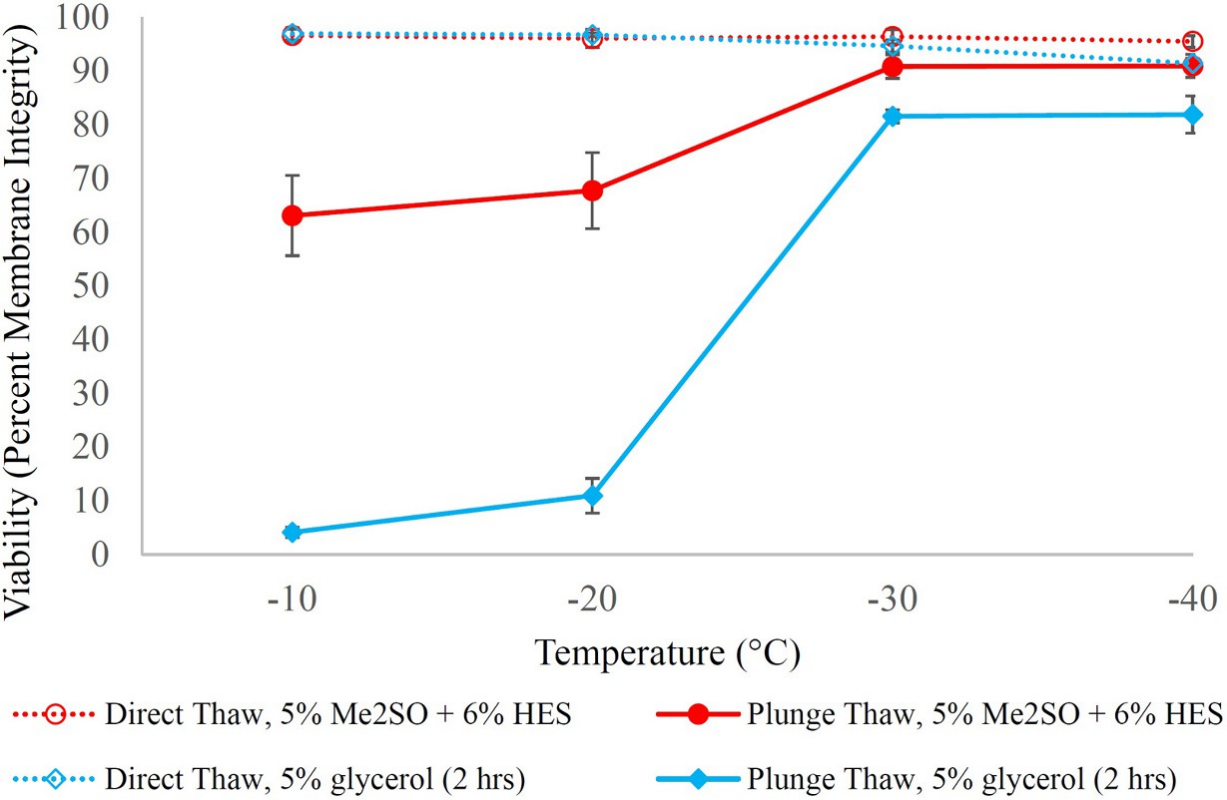
The two best protocols reported for H9c2 cryopreservation with and without Me_2_SO. One protocol uses Me_2_SO as the permeating cryoprotectant; cells were first incubated with 5% Me_2_SO + 6% HES for 15 minutes at 0°C. Extracellular ice was nucleated at −5°C, then cells were kept at that temperature for 3 minutes to allow the heat of fusion to be released. Cells were subsequently cooled at 1°C/min to −40°C (or to −30°C). The other protocol is Me_2_SO free; cells were incubated with 5% glycerol for two hours at room temperature. After ice nucleation at −5°C and keeping cells at that temperature for 3 minutes, cells were cooled at 1°C/min to −40°C (or to −30°C). Data points are the mean of independent experiments (at least 3 repeats) and error bars indicate standard deviation.

Next, the best protocols, which resulted in the highest membrane integrity were compared. Fig 5 shows the two successful cryopreservation protocols (with and without Me_2_SO) for H9c2 cells in suspension. The highest membrane integrity for cryopreservation of H9c2 in suspension was achieved in the presence of 5% Me_2_SO + 6% HES (Fig 5, red lines), which was 90.9 ± 2.1% when cells were plunged into liquid nitrogen at −40°C (or 90.8 ± 2.2% when cells were plunged into liquid nitrogen at −30°C). It is concluded that the best protocol to cryopreserve H9c2 cells in suspension involves incubating cells in the presence of 5% Me_2_SO + 6% HES (for 15 minutes at 0°C), nucleating extracellular ice at −5°C, holding for 3 minutes, then cooling at a rate of 1°C/min to either −30°C or −40°C before plunging and storing in liquid nitrogen. However, if a Me_2_SO-free cryopreservation protocol is desired, then cells can be incubated in 5% glycerol for two hours at room temperature (Fig 5, blue lines) before cooling, nucleating ice at −5°C, holding for 3 minutes, then cooling cells at a rate of 1°C/min to either −30°C or −40°C before plunging and storing them in liquid nitrogen.

### Effect of cryoprotectant removal on membrane integrity

**Fig 6:**
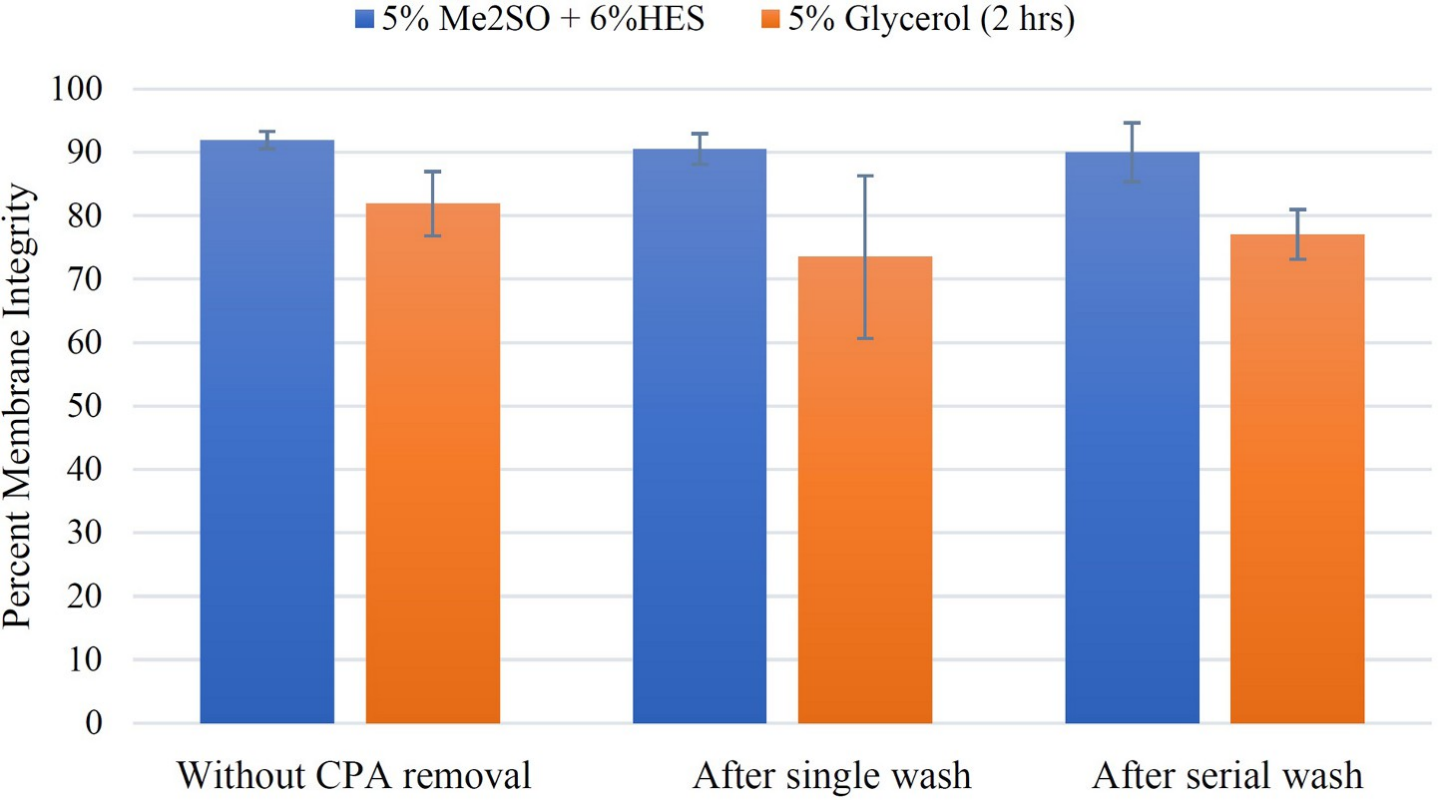
Effect of different methods of cryoprotectant removal on membrane integrity (relative viability). H9c2 suspension was cooled at 1°C/min to –40°C in the presence of either 5% Me_2_SO + 6% HES (incubated for 15 minutes at 0°C) or 5% glycerol (incubated for two hours at room temperature). Cells were then plunged into liquid nitrogen from –40°C, kept in liquid nitrogen for at least half an hour and then thawed (at 37°C) rapidly. Membrane integrities were then calculated based on trypan blue exclusion method i) immediately after thaw (without cryoprotectant removal); ii) after cryoprotectant (CPA) removal with either single or serial wash. Membrane integrity values for single and serial wash samples are the ratios of viable (TB-excluding) cells to total cells in the sample (after centrifugation). Data points are the mean of independent experiments (at least 3 repeats) and error bars indicate standard deviation.

Membrane integrity assessments for all graded freezing experiments that are mentioned above were performed immediately after thaw without cryoprotectant removal. However, post-thaw cell cultures and functional assessments often require cells without cryoprotectants. Consequently, we assessed membrane integrities for the two protocols developed in the previous section (5% Me_2_SO + 6% HES loaded for 15 minutes at 0°C and 5% glycerol loaded for two hours at room temperature) after cryoprotectant removal by one of two methods: single wash or serial wash (Fig 6). In a separate experiment, H9c2 suspension was incubated with 5% Me_2_SO + 6% HES at 0°C for 15 minutes, extracellular ice nucleated at −5°C, held for 3 minutes, then cooled at 1°C/min to −40°C, and plunged into liquid nitrogen at −40°C. After at least 30 minutes in liquid nitrogen, cells were thawed and assessed for membrane integrity by trypan blue *i*) immediately after thaw (without cryoprotectant removal), *ii*) after single wash, and *iii*) after serial wash. The membrane integrity without cryoprotectant removal was 91.9 ± 1.4% and membrane integrities were 90.5 ± 2.4% and 90.0 ± 4.6% after single wash and serial wash, respectively.

Membrane integrities after single wash or after serial wash were not statistically different from the immediate post-thaw membrane integrity (p = 0.08 and p = 0.3, respectively). Membrane integrity values for single wash and serial wash were also not statistically different from each other (p = 0.4). Table 4 indicates all p values for membrane integrity results before and after cryoprotectant removal (with different removal methods) when 5% Me_2_SO + 6% HES was used.

**Table 4:** p values for membrane integrities without cryoprotectant removal, and after different methods of cryoprotectant removal when 5% Me_2_SO + 6% HES was the cryoprotectant.

5% Me_2_SO + 6% HES	No CPA^*^ removal	After single wash	After serial wash
No CPA^*^ **removal**	-	p = 0.08	p = 0.3
**After single wash**	p = 0.08	-	p = 0.4
**After serial wash**	p = 0.3	p = 0.4	-

^*^ CPA stands for cryoprotectant agent.

In another experiment, H9c2 cells in suspension were incubated with 5% glycerol for two hours at room temperature, ice nucleated at −5°C, held for 3 minutes, cooled at 1°C/min to −40°C, then plunged into liquid nitrogen at −40°C. After at least 30 minutes in liquid nitrogen, cells were thawed and membrane integrity was assessed by trypan blue *i*) immediately after thaw (without cryoprotectant removal), *ii*) after single wash, and *iii*) after serial wash. Immediate after-thaw membrane integrity was 81.9 ± 5.1%. Membrane integrity after single wash was 73.5 ± 12.8%, and after serial wash was 77.1 ± 3.9%. According to p values (Table 5), all these results were not significantly different.

**Table 5:** p values for membrane integrities without cryoprotectant removal, and after different methods of cryoprotectant removal when 5% glycerol (two hours) was the cryoprotectant.

5% glycerol (2 hours)	No CPA^*^ removal	After single wash	After serial wash
No CPA^*^ **removal**	-	p = 0.15	p = 0.22
**After single wash**	p = 0.15	-	p = 0.36
**After serial wash**	p = 0.22	p = 0.36	-

^*^ CPA stands for cryoprotectant agent.

**Fig 7:**
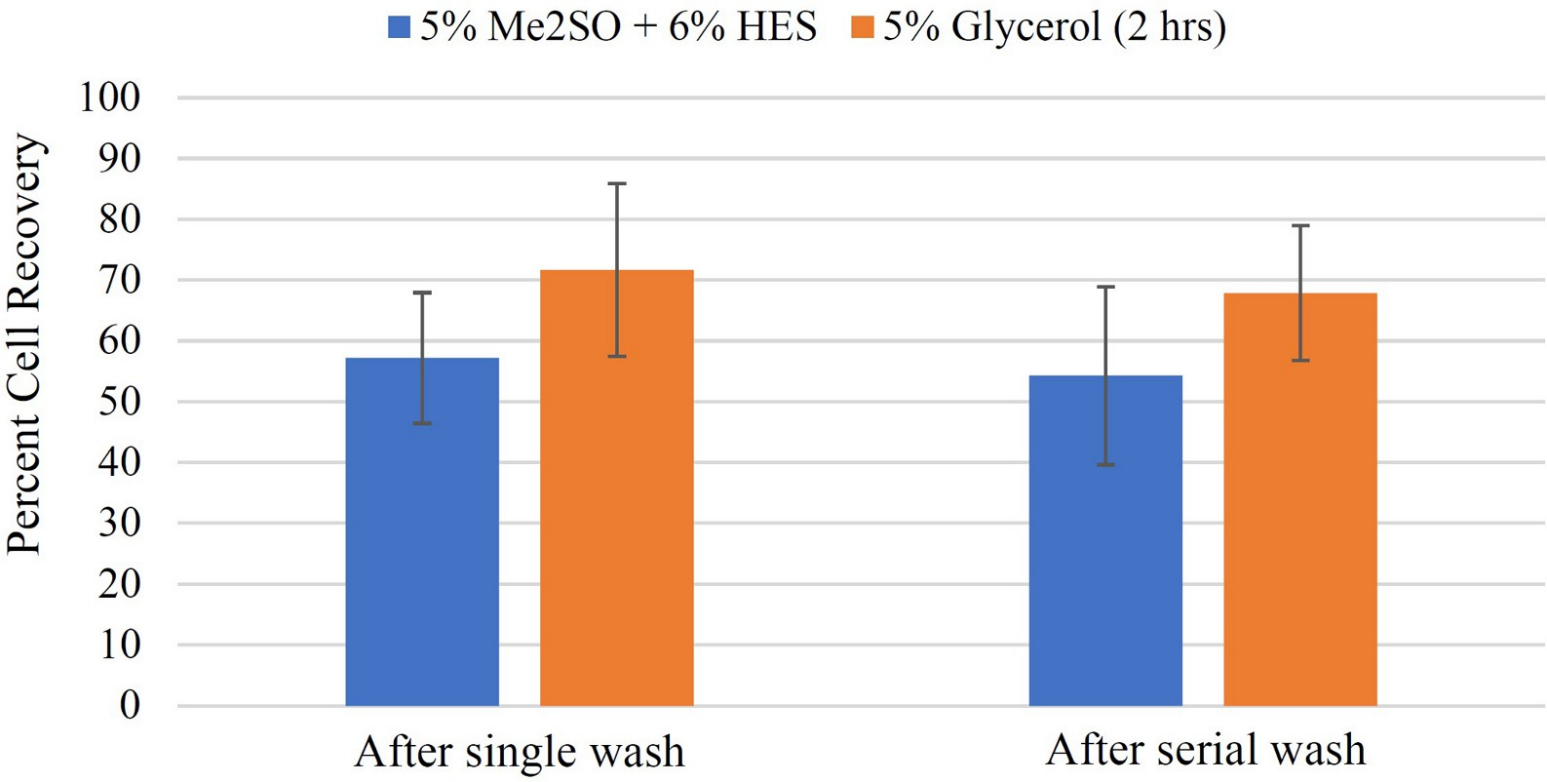
Effect of different methods of cryoprotectant removal on cell recovery. H9c2 suspension was cooled at 1°C/min to –40°C in the presence of either 5% Me_2_SO + 6% HES (incubated for 15 minutes at 0°C) or 5% glycerol (incubated for two hours at room temperature). Cells were then plunged into liquid nitrogen from –40°C, kept in liquid nitrogen for at least half an hour and then thawed (at 37°C) rapidly. Membrane integrities were calculated based on trypan blue exclusion method i) immediately after thaw (without cryoprotectant removal); ii) after cryoprotectant (CPA) removal with either single or serial wash. Cryoprotectant removal followed by centrifugation may remove cells from the sample, which makes it important to know the recovery of the remaining cells in the sample. Thus, cell recovery was calculated. Cell recovery is the ratio of the remaining membrane-intact (TB-excluding) cells in each sample after cryoprotectant removal and centrifugation to the membrane-intact (TB-excluding) cells in the sample immediately after thaw. Data points are the mean of independent experiments (at least 3 repeats) and error bars indicate standard deviation.

It is important to point out that the viabilities measured in Fig 6 were calculated based on relative viability, which is the ratio of live cells in the sample to the total cells in the sample; however, damaged cells (dead cells) may be lost during washing and centrifugation inflating the relative recovery of the sample. To address this point, we calculated the cell recovery to have a better understanding of viability of the samples (Fig 7). Cell recovery was calculated according to Eq 3.


$$\%\;\text{Cell}\;\text{recovery}\;\text{upon}\;\text{cryoprotectant}\;\text{removal}\;=\frac{\text{TB-excluding}\;\text{cells}\;\text{after}\;\text{cryoprotectant}\;\text{removal}}{\text{TB-excluding}\;\text{cells}\;\text{immediately}\;\text{after}\;\text{thaw}}\times 100\%$$ (3)

For 5% Me_2_SO + 6% HES, cell recoveries were 57.2 ± 10.7% and 54.3 ± 14.6% after single wash and serial wash, respectively. Single wash and serial wash showed comparable results based on p value (p = 0.1). For glycerol, cell recoveries were 71.7 ± 14.2% and 67.9 ± 11.1% after single and serial wash, respectively. p value (p = 0.1) shows that for glycerol too, either single or serial wash could be applied. Single wash was chosen as the post-thaw cryoprotectant removal method for functional assessment experiments because single wash was simpler and required shorter time compared to serial wash.

### Functional assessment

**Fig 8:**
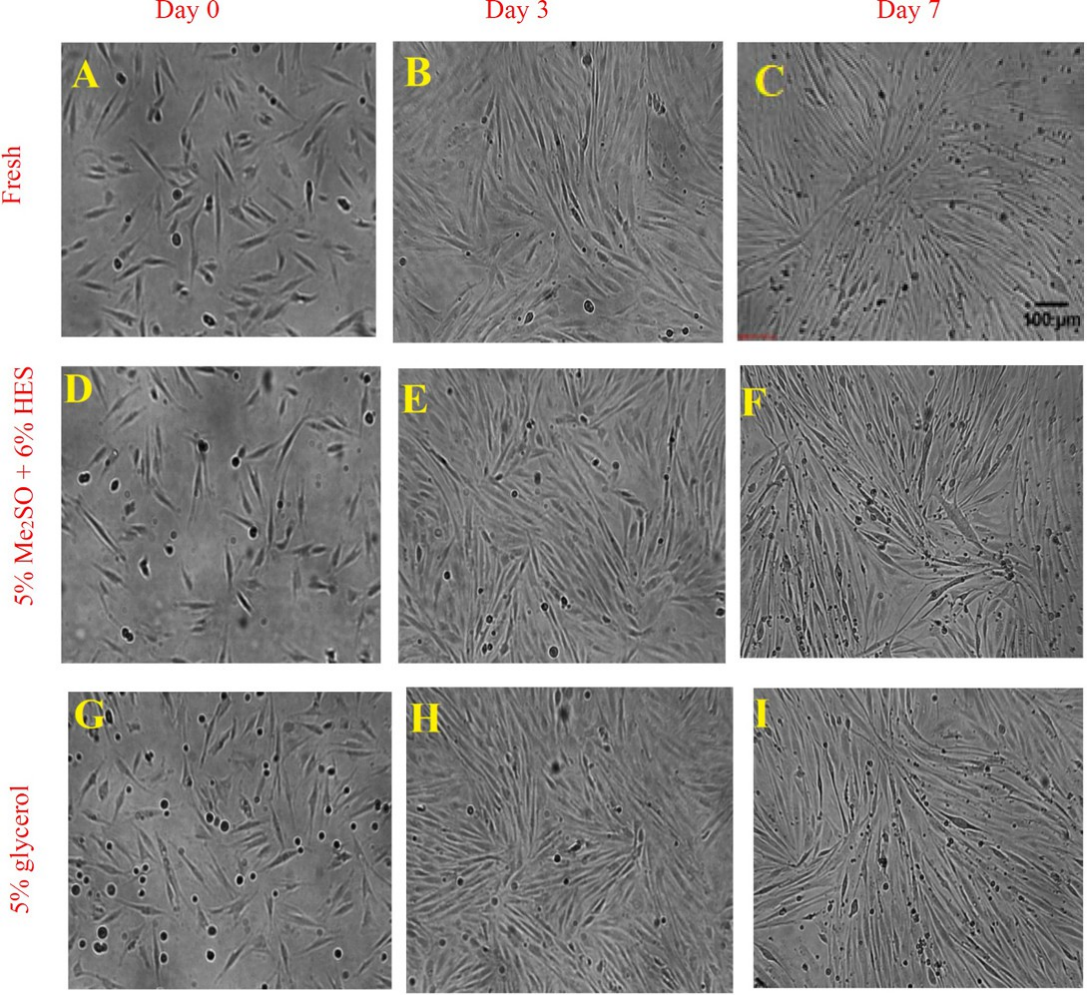
Phase contrast images showing differentiation of H9c2 myoblasts to myotubes. Fresh and frozen (by the two best protocols) and thawed H9c2 myoblasts were seeded in 24-well plates in complete culture medium (supplemented with 10% FBS). The next day, the serum concentration was reduced to 1% in culture medium. All-trans retinoic acid (ATRA) was supplemented in the culture medium in addition to serum deprivation. Pictures show the process of differentiation from day 0 up to day 7 of serum deprivation and ATRA stimulation. Figures A, B, and C represent fresh (unfrozen H9c2 cells); figures D, E, and F represent frozen and thawed H9c2 cells that have been frozen in 5% Me_2_SO + 6% HES; and figures G, H, and I represent frozen and thawed H9c2 cells that have been frozen in 5% glycerol with two hours loading.

Membrane-intact cells are not necessarily always alive and functional. Thus, it was required to assess that the cryopreserved cells are not only membrane-intact, but also exhibit the characteristics of H9c2 myoblasts. Some of the H9c2 characteristics to be assessed were: *i*) ability to differentiate to cardiac myotubes; *ii*) increased mitochondrial mass during differentiation to cardiac myotubes; and *iii*) cardiac troponin I expression during differentiation to myotubes. We cultured fresh (unfrozen) H9c2 (control) or cryopreserved Hc92 cells (in either 5% Me_2_SO + 6% HES in complete growth medium incubated for 15 minutes at 0°C or 5% glycerol in complete growth medium incubated for two hours at room temperature). Fig 8 shows that both fresh and frozen (with either protocol) H9c2 mononucleated myoblasts differentiated into cardiac multinucleated myotubes.

**Fig 9:**
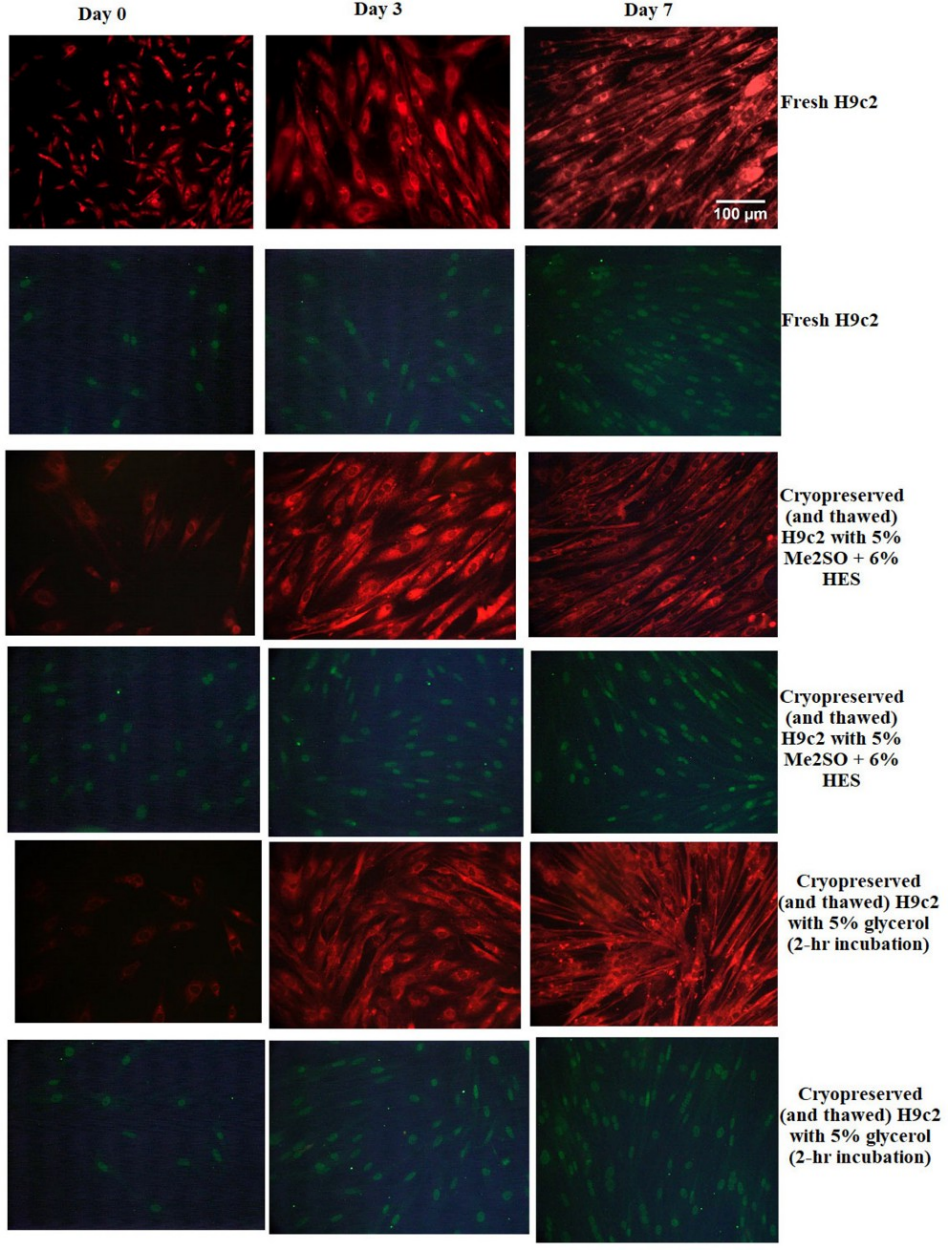
Mitochondrial mass and troponin I expression for H9c2 cells. Fresh and frozen (with the two best protocols) and thawed H9c2 cells were seeded in 24-well plates in complete culture medium. (Supplemented with 10% FBS). The next day, the serum concentration was reduced to 1% in culture medium. All-trans retinoic acid (ATRA) was supplemented in the culture medium in addition to serum deprivation. Mitochondrial mass (red pictures) and troponin I (green pictures) expression increased during differentiation to cardiac myotubes after 3 and 7 days of serum deprivation and ATRA stimulation of fresh H9c2 cells, Hc92 cells cryopreserved in 5% Me_2_SO + 6% HES (and then thawed), and Hc92 cryopreserved in 5% glycerol (two hours loading; and then thawed).

Fig 9 shows the process of mitochondrial mass increase and troponin I expression from day 0 (without serum deprivation and ATRA stimulation) to 3 and 7 days of serum deprivation and ATRA stimulation showing that H9c2 differentiated to cardiac myotubes. As seen in Fig 9, the formation of multinucleated myotubes was observed at day 7 for fresh and frozen cells; in addition, expression of troponin I increased by formation of multinucleated myotubes at day 7 for fresh and frozen cells.

## Discussion

Cells experience different stresses during cryopreservation. The most fatal stresses experienced by cells happen during cooling to sub-zero intermediate temperatures (−15 to −60°C) and warming from these temperatures, which means that cells endure this situation twice [95]. Therefore, the first step in cryopreservation studies is evaluating cell response to freeze/thaw stress, which is essential in order to design and optimize cryopreservation protocols [103]. The main objective of this study was to systematically optimize cryopreservation protocols for H9c2 cells in suspension by applying a graded freezing procedure, and demonstrate that H9c2 cells remain viable, and maintain their functionality after cryopreservation. Graded freezing is a useful tool to distinguish cooling injuries that occur during cooling to the intermediate sub-zero temperatures (slow cooling injuries), from those that happen during cooling from each intermediate temperature to liquid nitrogen temperature (rapid cooling injuries); which was first used by McGann in 1979 [23], and has been applied in many studies so far [26,58,77,93,94,103,106–109]. During graded freezing, cells lose sufficient intracellular water over a range of temperatures during initial cooling, so that intracellular ice formation can be prevented during further cooling to −196°C. The decline in graded-freezing direct-thaw membrane integrity values in the absence of cryoprotectants confirms that H9c2 cells are vulnerable to slow cooling injuries. Likewise, if the cells are not slow-cooled to sufficiently low sub-zero temperatures in order to lose sufficient intracellular water before plunge into liquid nitrogen (to prevent intracellular ice formation, which is lethal [7,110]), they are similarly susceptible to rapid cooling injuries. Hence, the presence of a permeating cryoprotectant is mandatory in cryopreservation of H9c2 cells in suspension because permeating cryoprotectant protects cells against slow cooling injuries and helps cells to tolerate lower sub-zero temperatures to lose sufficient water to minimize the intracellular ice formation when cells are plunged into liquid nitrogen. Accordingly, a permeating cryoprotectant can improve both direct-thaw and plunge-thaw membrane integrities. The H9c2 manufacturer recommends 5% Me_2_SO as the permeating cryoprotectant to cryopreserve this cell type. However, we explored addition of a non-permeating cryoprotectant (HES) to this mixture and found out that HES provided even more protection against intracellular ice formation when cells were plunged into liquid nitrogen; therefore, the plunge-thaw membrane integrities were improved by addition of HES (Table 1). We similarly explored application of another permeating cryoprotectant (glycerol) as an alternative to Me_2_SO suitable for situations in which the presence of Me_2_SO is undesired. Glycerol experiments were initiated with incubating cells in 5% (w/w) glycerol for one hour at room temperature. However, the direct-thaw results (it is preferred that direct-thaw values stay the same throughout the slow cooling; yet, they decreased slightly during cooling down to −40°C) showed that 5% (w/w) glycerol or one hour incubation time might be insufficient in conferring complete protection against slow cooling injuries, and that cells could possibly benefit from better permeation of glycerol by either increasing the glycerol concentration and/or the incubation time at room temperature. For this reason, we next increased the concentration of glycerol to 10% (w/w) (incubated for one hour at room temperature). Comparing 5% and 10% glycerol direct-thaw data, though, it was shown that increasing the concentration of glycerol from 5% to 10% (w/w) did not improve the cell protection against slow cooling injuries significantly (Table 2). Increasing the incubation time for 5% glycerol from one hour to two hours, however, improved the direct-thaw as well as plunge-thaw membrane integrities (Table 3) significantly. It was shown in this study that providing longer time (two hours at room temperature) for glycerol to permeate cells improves the ability of glycerol to protect cells against slow cooling injuries through colligative effects. Hence, 5% glycerol with two hours incubation at room temperature was chosen as the best protocol for glycerol. Accordingly, two optimal cryopreservation protocols were determined. The main outcome of this study is the development of two systematic and well-optimized cryopreservation protocols: *i*) one using a combination of Me_2_SO and HES, and *ii*) one using glycerol. Each of these protocols can be helpful based on the application of interest; for example, when Me_2_SO is not problematic, time really matters, and/or the highest viability is desired, the first protocol seems to be more appropriate. On the other hand, when the application needs to eliminate Me_2_SO (and this aspect is more important than time), the second protocol (glycerol) seems to be more suitable. Both protocols resulted in high post-thaw viabilities for H9c2 myoblasts and maintained their functionality compared to fresh cells. After determining two optimal cryopreservation protocols, cells cryopreserved with these protocols (and then thawed) were cultured to differentiate and form cardiac myotubes. Formation of cardiac myotubes was confirmed by observation of *i*) cell morphology change (change from mononucleated spindle shape to large multinucleated tubular shape), *ii*) mitochondria morphology and mass change (increase in mitochondrial mass, showing more mitochondria staining), and *iii*) increasing expression of cardiac troponin I from day 1 to day 7 of serum reduction and ATRA stimulation, which was in agreement with previous studies [69,70,82,85,111]. The expression of troponin I was stronger when more myoblasts were differentiating to cardiac myotubes from day 1 to day 7. It can be mentioned that the increase in Mito Tracker Red fluorescence (mitochondrial mass) came about concurrently with the increase in troponin I, which confirmed formation of more cardiac myotubes during day 1 to day 7 and agrees with what Comelli at al. [85] reported.

It was observed that cryopreserved H9c2 cells (with either protocol) showed comparable results to the unfrozen sample. Cryopreserved H9c2 myoblasts showed the ability to differentiate to cardiac myotubes after 7 days of serum reduction and ATRA stimulation, and expressed the morphology change, increase in mitochondrial mass, and troponin I expression comparable to fresh controls. It is worth mentioning that troponin I is a cytoplasmic protein, and its expression is expected to be observed inside the cytoplasm [112–114]; however, in this study, the localization of troponin I seemed to be around the nucleus. This is because H9c2 cells lack a fully developed sarcomere apparatus, which is the structure responsible for muscle contraction, since H9c2 cells are a myoblast cell line and not primary cardiomyocytes [115].

## Conclusion

We applied graded freezing to investigate and optimize the response of H9c2 cells to freeze/thaw processes in the absence of cryoprotectants and in the presence of different cryoprotectants. Important factors that affect the cell response to freeze/thaw were optimized. One of these key factors was the different types and concentrations of cryoprotectants. The cryoprotectants studied were: *i*) 5% (w/w) Me_2_SO; *ii*) 5% (w/w) Me_2_SO + 6% (w/w) HES; *iii*) 5% (w/w) glycerol; and *iv*) 10% (w/w) glycerol. Cryoprotectants were added to the H9c2 complete growth medium. The next important factor explored was cryoprotectant incubation time and temperature. Cells were incubated with 5% Me_2_SO, or 5% Me_2_SO + 6% HES for 15 minutes at 0°C; while they were incubated with glycerol at room temperature for either one or two hours. The next important factor was purposeful ice nucleation at a specific temperature to prevent variable results and minimize the probability of intracellular ice formation by relieving the supercooling. In this study, −5°C was chosen as the temperature at which the extracellular ice was induced. Another important factor that affects the cell response to freeze/thaw is cooling profile. In this study, an interrupted slow cooling protocol was applied to distinguish the injuries incurred by H9c2 cells during slow cooling from those that were incurred during fast cooling (plunge into liquid nitrogen). Cells were cooled at 1°C/min to different sub-zero temperatures (−10, −20, −30, and −40°C). At each of these intermediate temperatures, some cells were thawed and their membrane integrities were examined (direct-thaw); while other cells were plunged into liquid nitrogen, stored for at least 30 minutes, and then thawed and their membrane integrities were examined (plunge-thaw). Membrane integrity viabilities for direct-thaw samples indicate the injuries that cells experience during slow cooling such as solute effects and cell shrinkage injuries; permeating cryoprotectants can protect cells against these injuries. In the case of decrease in direct-thaw results from −10°C to −40°C, the concentration of permeating cryoprotectant and/or the incubation time to allow permeation should be increased. The further drop in membrane integrity for plunge-thaw samples compared to direct-thaw samples indicates rapid cooling injuries known to be caused by intracellular ice formation; non-permeating cryoprotectants can protect cells against these injuries. In this study, addition of HES improved the plunge-thaw results. Another key factor investigated in this study was plunge temperature. It was shown here (based on both optimal protocols) that cooling cells at 1°C/min to either −30°C or −40°C, and then plunging them into liquid nitrogen resulted in better post-thaw membrane integrities; hence, either −30°C or −40°C could be chosen as the best plunge temperature (−40°C was chosen here as the plunge temperature for further post-thaw functionality assessment). The last important factor was the cryoprotectant removal procedure because cryoprotectant removal is necessary for functional assessment. Two methods (single wash and serial wash) were applied for cryoprotectant removal after thaw to investigate their affects on membrane integrity. Results shows that both removal methods were comparable, and their membrane integrities (relative viabilities) were comparable to those achieved without cryoprotectant removal. Calculation of cell recovery showed that some cells were lost during cryoprotectant removal and centrifugation, but that the cell recoveries were comparable for the two different cryoprotectant removal methods for each protocol. Single wash was chosen (over serial wash) for cryoprotectant removal after thaw because it was faster and required less time. Two comprehensive cryopreservation protocols were provided for H9c2 cells in this study. The first protocol applies Me_2_SO and HES and is suitable for those applications in which time matters, the presence of Me_2_SO is not an issue, and the highest viabilities are desired. Incubating H9c2 in suspension with 5% Me_2_SO + 6% HES in complete growth medium for 15 minutes at 0°C, nucleating extracellular ice at −5°C, holding cells at −5°C for 3 minutes to allow the heat of fusion to be released, cooling cells at 1°C/min to −40°C, and plunging cells into liquid nitrogen at −40°C yielded immediate post-thaw membrane integrities of 90.9 ± 2.1% based on SYTO 13/GelRed by flow cytometry. The second protocol applies glycerol as a substitute for Me_2_SO; this protocol is suitable for applications in which use of Me_2_SO is undesirable and the longer incubation time and slightly lower viabilities are acceptable. Incubating H9c2 in suspension with 5% glycerol for two hours at room temperature, nucleating extracellular ice at −5°C, holding cells at −5°C for 3 minutes to allow the heat of fusion to be released, cooling cells at 1°C/min to −40°C, and plunging cells into liquid nitrogen at −40°C yielded immediate post-thaw membrane integrities of 81.8 ± 3.5% based on SYTO 13/GelRed by flow cytometry. Then, fresh (unfrozen) H9c2 as well as H9c2 cryopreserved with both protocols (that were then thawed and had their cryoprotectants removed by single wash) were cultured in complete medium for 24 hours. After that, serum concentration was decreased and ATRA was supplemented in the medium (of each sample) to allow H9c2 cells to differentiate to cardiac myotubes. Differentiation of either fresh or cryopreserved (and thawed) H9c2 was confirmed by *i*) morphology change (irregular mononucleated spindle shape cells changed to large multinucleated tubular cells); *ii*) the expression of cardiac marker (troponin I); and *iii*) increase in mitochondrial mass. Both protocols could successfully preserve H9c2 cell viability and functionality after thaw. These protocols can provide on demand and cost-effective access to high quality H9c2 cells.

## Supporting information

S1 File (PDF)
