# Supplementary material for: Systematic cryopreservation study of cardiac myoblasts in suspension
Source: PLoS One. 2024 Mar 6;19(3):e0295131. doi: 10.1371/journal.pone.0295131 (PMC10917286; doi:10.1371/journal.pone.0295131)
Supplement: S1 File — (PDF) [file pone.0295131.s001.pdf]

Supplementary Information S1 File:

Comprehensive cryopreservation study of H9c2 cells in suspension

Elham Ashrafi[1], Milica Radisic[3,4], Janet A. W. Elliott[1,2*]

[1]Department of Chemical and Materials Engineering, University of Alberta, Edmonton, Alberta, Canada

[2]Department of Laboratory Medicine and Pathology, University of Alberta, Edmonton, Alberta, Canada

[3]Institute of Biomedical Engineering, University of Toronto, Toronto, Ontario, Canada

[4]Department of Chemical Engineering and Applied Chemistry, University of Toronto, Toronto, Ontario, Canada

[*]Corresponding author:

Email: janet.elliott@ualberta.ca (JAWE)

Although the H9c2 manufacturer had recommended to re-culture attached cells when they reach 50–70% surface coverage, in some experiments, cells were overgrown (around 85% surface coverage); this triggered designing of some experiments with the aim to compare the cell response to freeze/thaw processes when they were overgrown versus when they were grown in the acceptable range (50–70% surface coverage). Experiments were designed to freeze/thaw H9c2 cells with 5% Me_2SO (dimethyl sulfoxide), with 5% Me_2SO+6% HES (hydroxyethyl starch), and with 5% glycerol (one hour incubation at room temperature) in two different situations (overgrown and acceptable growth).

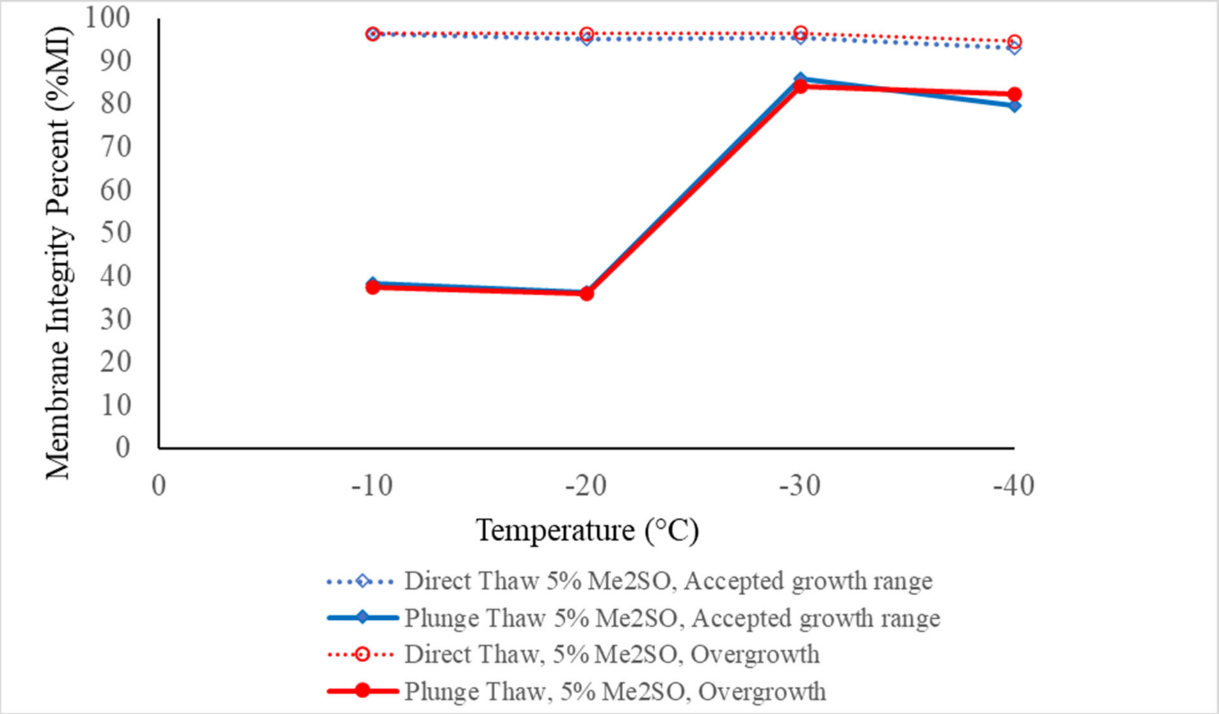

Figure S1. Comparison of H9c2 response when it was overgrown vs. within accepted growth range when 5% Me_2SO was used.

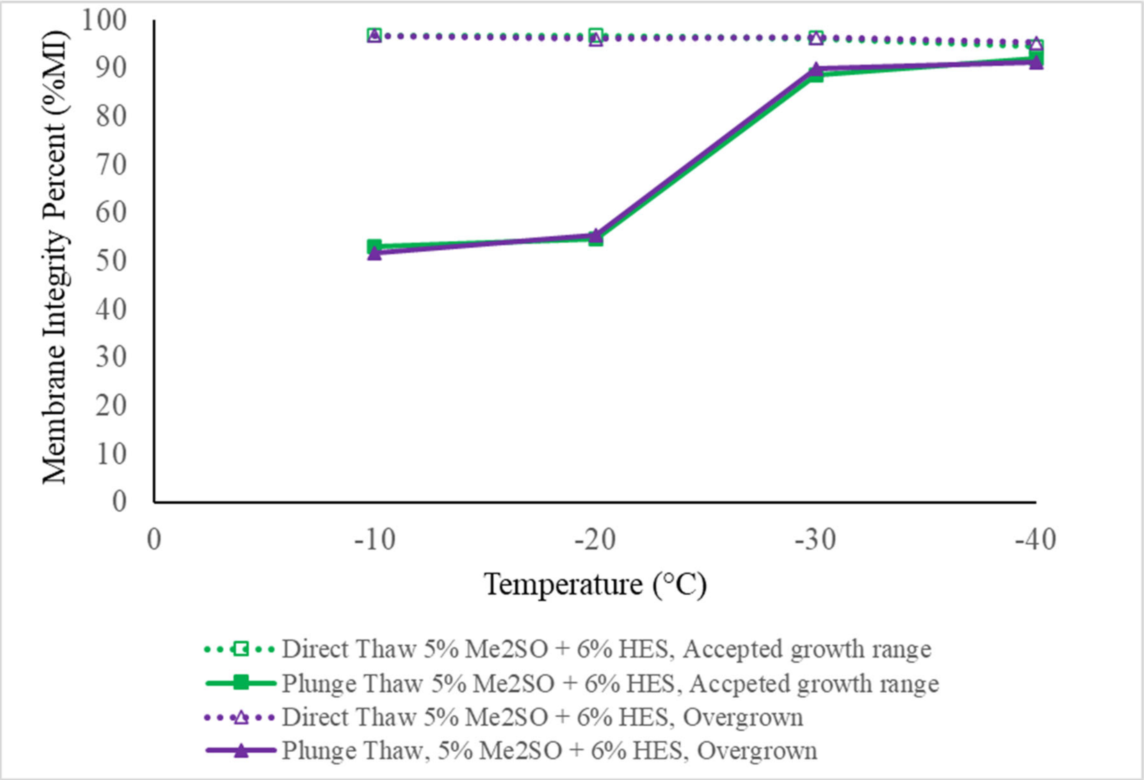

Figure S2. Comparison of H9c2 response when it was overgrown vs. within accepted growth range when 5% Me2SO + 6% HES was used.

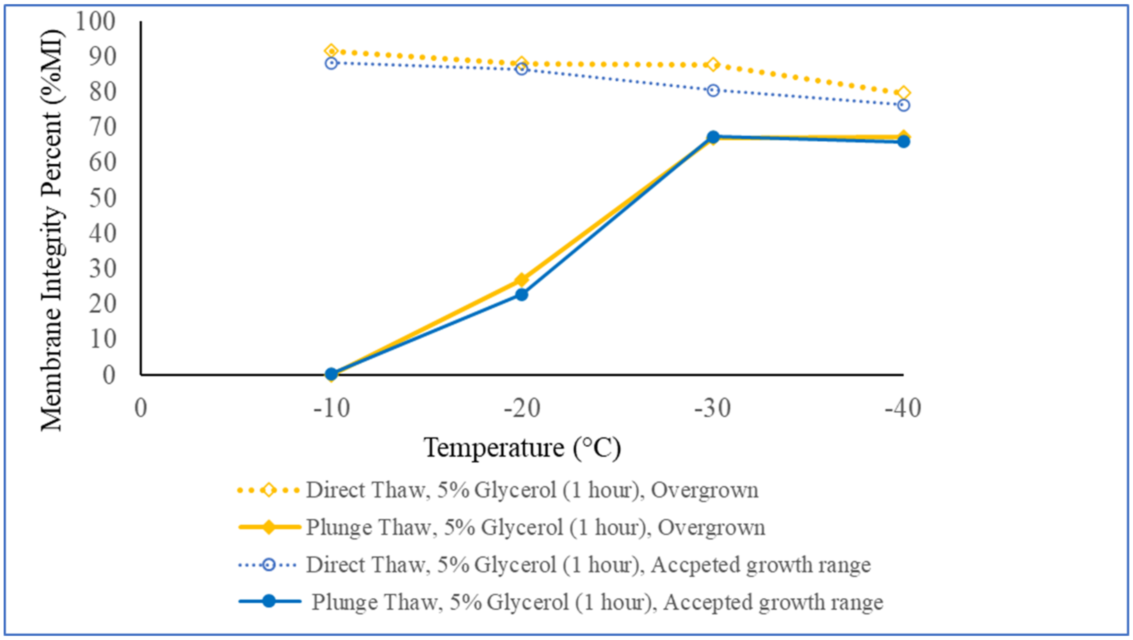

Figure S3. Comparison of H9c2 response when it was overgrown vs. within accepted growth range when 5% glycerol (one hour) was used.

Results from Figures S1–S3 indicate that overgrowth (over 70% surface coverage) did not affect the H9c2 response to freeze/thaw processes for all different cryoprotectants; thus, all these data were combined (all acceptable growth samples and overgrown samples), and error bars were calculated for Figures 3 and 4 in the main text.

To investigate whether the passage number affects the H9c2 cell type response to cooling/warming procedures, we designed several experiments to freeze/thaw H9c2 cells with 5% Me_2SO, or with 5% Me_2SO+6% HES at various passage numbers (we chose 4, 7, and 10) to see how different their responses were. Figures S4 and S5 show responses of H9c2 cells at different passage numbers when cryopreserved with 5% Me_2SO and with 5% Me_2SO+6% HES.

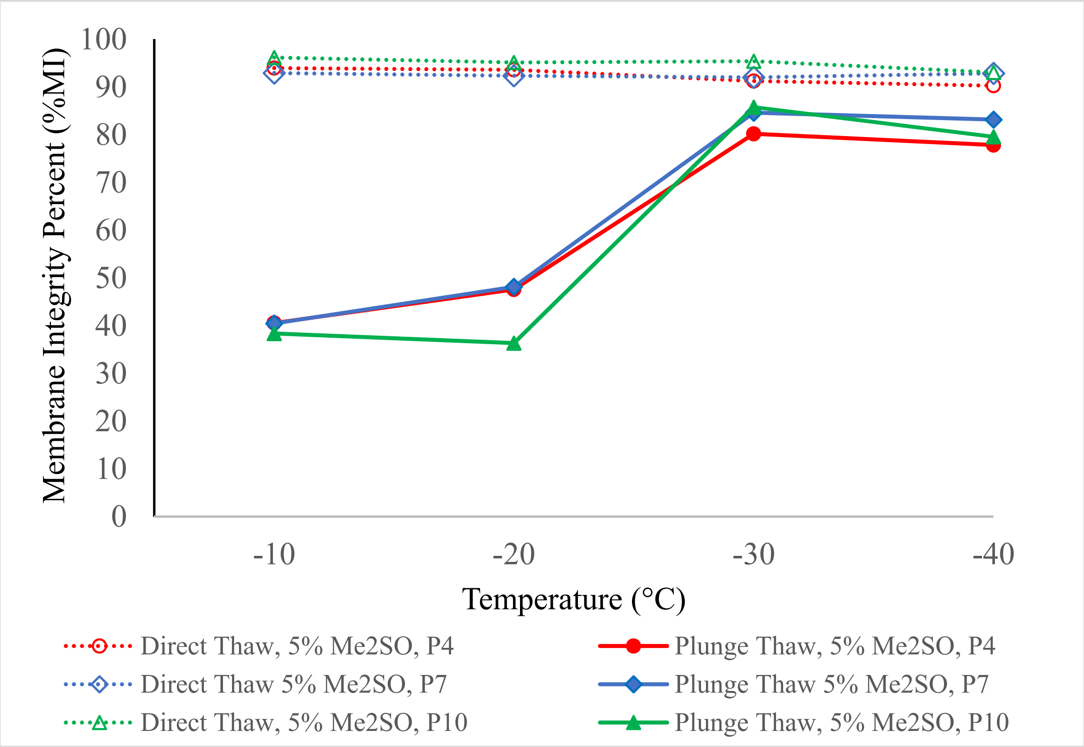

Figure S4. Effect of passage number on H9c2 response when 5% Me2SO was used.

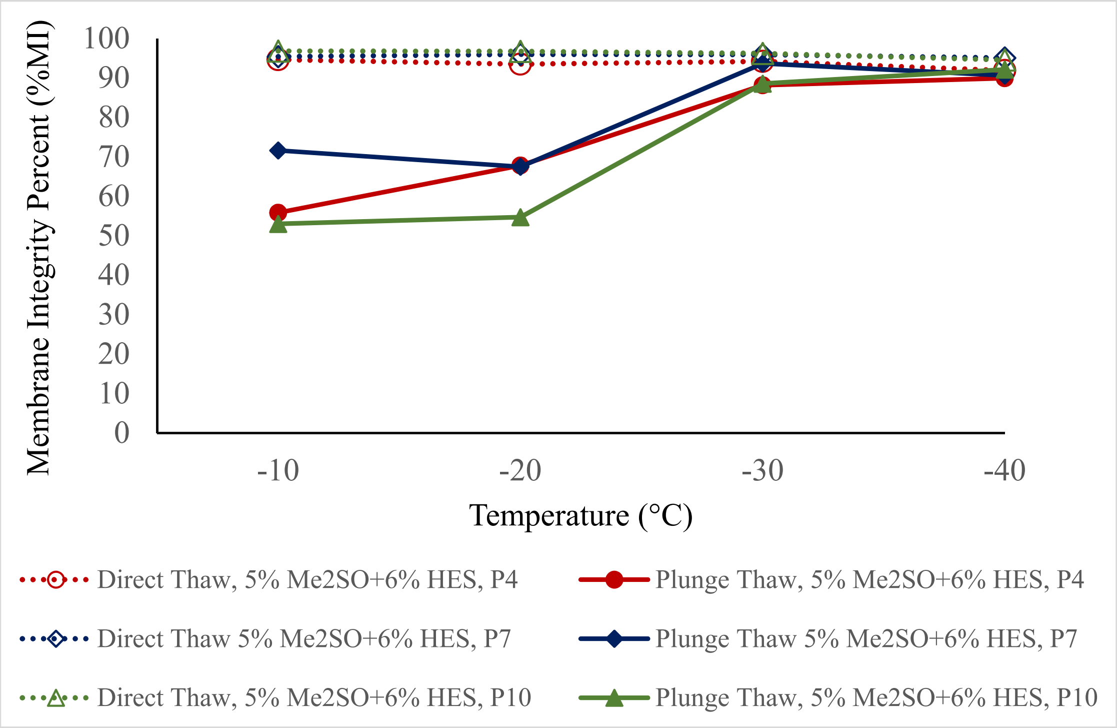

Figure S5. Effect of passage number on H9c2 response when 5% Me2SO + 6% HES was used.

Figure S6 shows H9c2 cryopreservation response at different passage numbers (passage 7, and 10) when cryopreserved with 5% glycerol (one hour incubation time at room temperature).

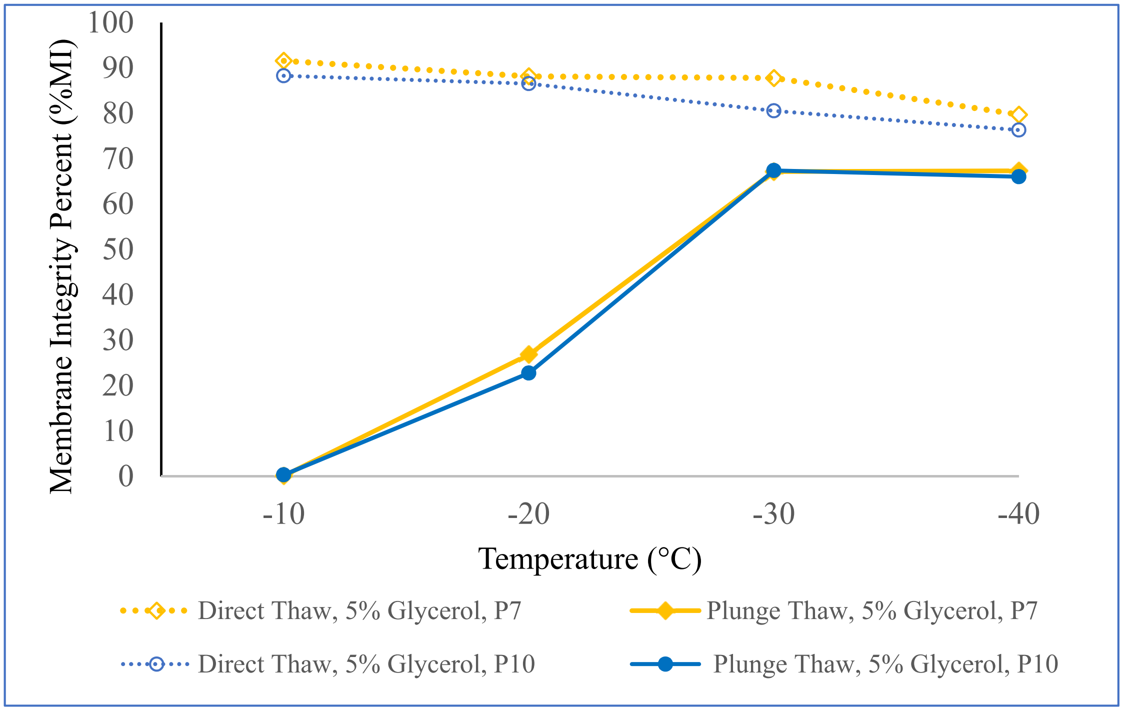

Figure S6. Effect of passage number on H9c2 response when 5% glycerol (one hour) was used.

Results from Figures S4–S6 show that passage number did not affect the freeze/thaw response for H9c2 cells; therefore, the passage numbers used during the whole project were from 2 to 10 and all these experiments at different passage numbers were combined, and error bars were calculated for Figures 3 and 4in the main text.

The cooling rate during plunge into liquid nitrogen from −40 °C was calculated. One thermocouple was located inside a sample glass tube in the methanol bath. This thermocouple was connected to a computer with a screen that monitors the temperature and time. When the sample reached to −40 °C, the sample was plunged into liquid nitrogen. Temperature change was monitored (with time); the data were collected in Excel and the graph was produced. This experiment was repeated three

times (Figure S7). A best fit was calculated based on a linear equation for temperatures between −40 °C and −180 °C. The average cooling rate calculated based on three repeats was 17.35 ± 2.15 °C/s which is 1041.0 ± 129.0 °C/min.

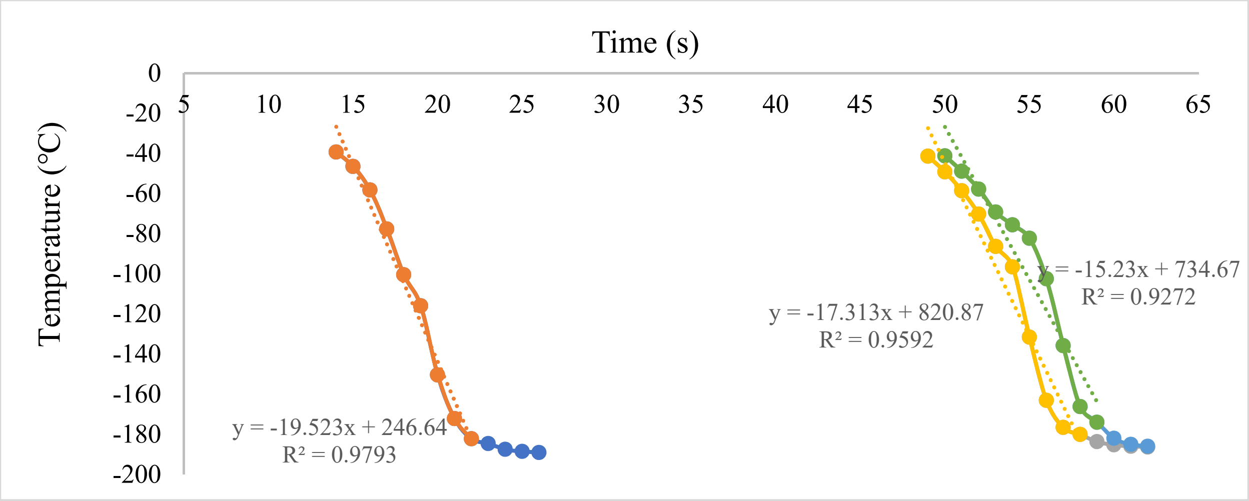

Figure S7. Temperature change for H9c2 samples that had been plunged into liquid nitrogen from −40 °C.

Similarly, the warming rates for direct-thaw and plunge-thaw for samples that were either directly thawed from −40 °C or thawed after plunging into liquid nitrogen from −40 °C (and stored for at least 30 minutes) were calculated. Figures S8 and S9 show these warming rates. The temperature range considered (for a linear equation) to calculate the warming rate for direct-thaw samples was −38°C to 20 °C. The warming rate for direct-thaw sample was 3.72 ± 0.54 °C/s which is 223.2 ± 32.4 °C/min (Figure S8). The temperature range considered (for a linear equation) to calculate warming rate for plunge-thaw samples was −150 °C to −10 °C. The warming rate for plunge-thaw sample was 23.3 ± 5.0 °C/s which is 1398 ± 300.0 °C/min (Figure S9).

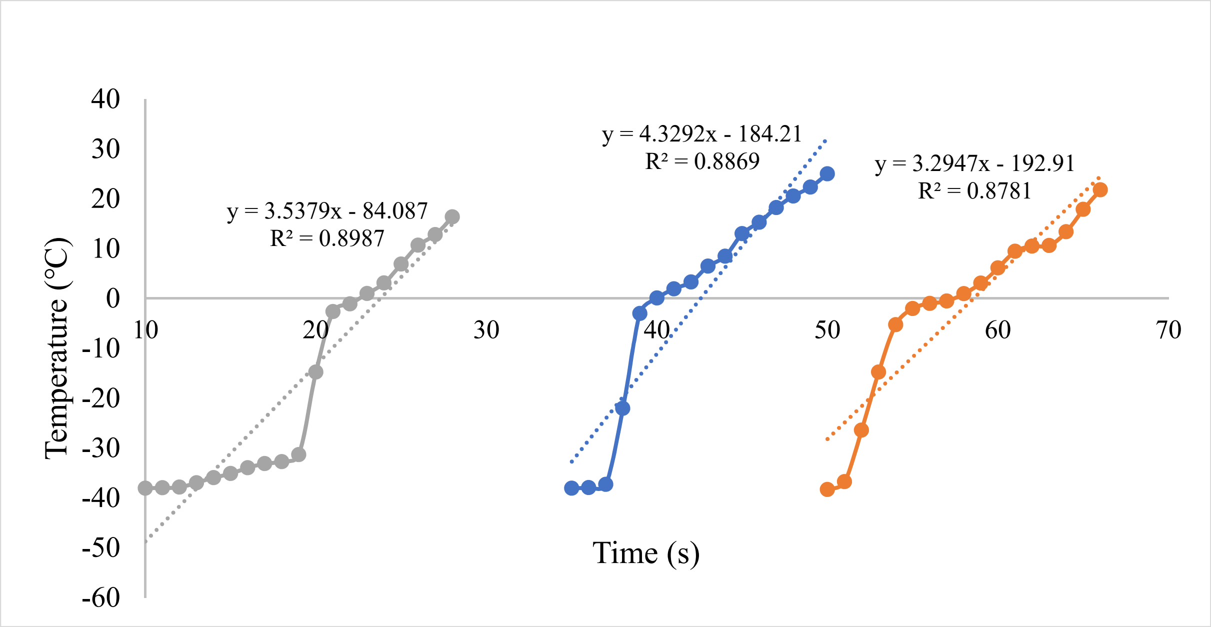

Figure S8. Temperature change for H9c2 samples that had been thawed from −40 °C.

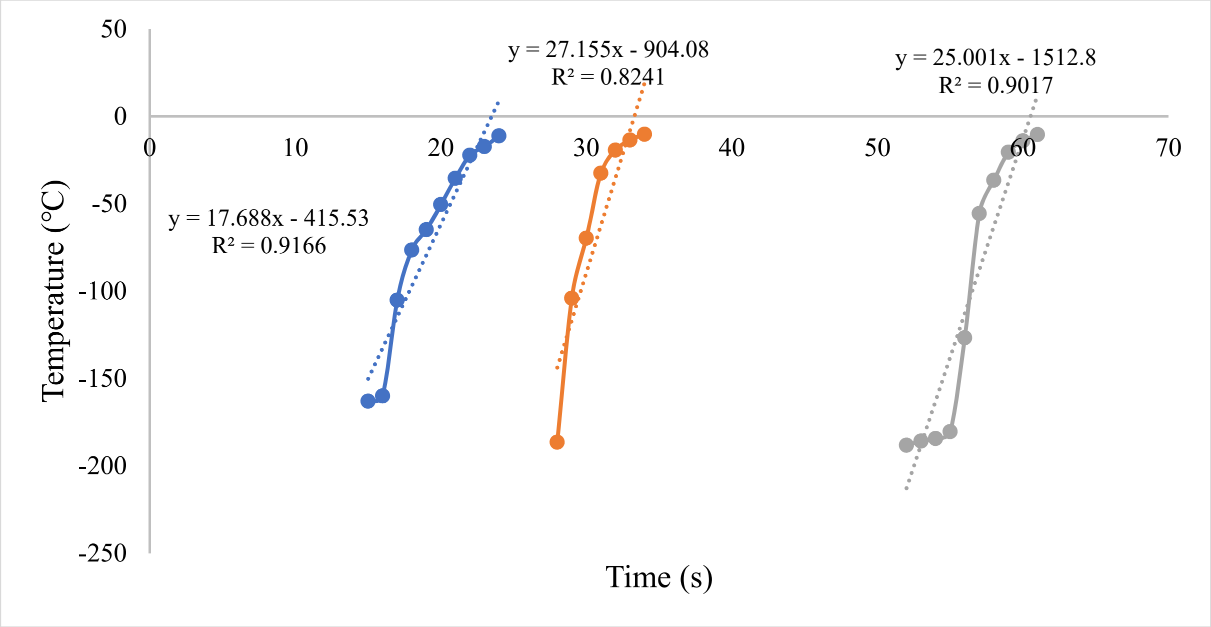

Figure S9. Temperature change for H9c2 samples that had been thawed from liquid nitrogen.
